# Identifying barriers and facilitators to implementing triage systems in low- and middle-income countries: A scoping review

**DOI:** 10.1371/journal.pgph.0007378

**Published:** 2026-09-29

**Authors:** Ghada Elhadari, Sarah Aly, Alexandria Brackett, Hani Mowafi

**Affiliations:** 1 Department of Emergency Medicine, Yale University, New Haven, Connecticut, United States of America; 2 Department of Emergency Medicine, Faculty of Medicine, Suez Canal University, Ismailia, Egypt; 3 Department of Emergency Medicine, Loma Linda University, Loma Linda, California, United States of America; 4 Harvey Cushing/John Hay Whitney Medical Library, Yale University, New Haven, Connecticut, United States of America; NYU Grossman School of Medicine: New York University School of Medicine, UNITED STATES OF AMERICA

## Abstract

More than 50% of mortality and one-third of disabilities associated with a range of medical and surgical conditions may be prevented by a well-established Emergency Care System (ECS). The World Health Organization (WHO) has developed a framework that defines the essential ECS functions, including facility-based triage. This scoping review aims to identify the barriers and facilitators to facility-based triage implementation in low- and middle-income countries (LMICs) within peer-reviewed literature and map them to the Consolidated Framework for Implementation Research (CFIR). We searched Scopus, Web of Science-Core Collection, Ovid MEDLINE, Embase, Cochrane CENTRAL, CINAHL, AfricaWide, and Global Health for original research articles, with one or more barriers or facilitators to triage implementation in an LMIC facility. Two reviewers independently screened studies in Covidence. A third reviewer resolved conflicts at each stage. Data was extracted in Covidence by one reviewer and verified by a second. We conducted thematic content analysis of barriers and facilitators and mapped them to the Consolidated Framework for Implementation Research (CFIR). We also conducted quality appraisals of each study using Joanna-Briggs Institute (JBI) tools and the Mixed Method Appraisal tool (MMAT). Our search yielded 1,586 articles, including 839 unique articles. After applying inclusion/exclusion criteria, we included 57 studies. 42 (73.7%) studies were mapped to the Innovation domain; 19 (33.3%) studies were mapped to the Outer Setting domain; 50 (87.8%) studies were mapped to the Inner Setting domain; 51 (89.5%) studies were mapped to the Characteristics of Individuals domain; 53 (93.0%) studies were mapped to the Implementation Process domain. Several barriers and facilitators to triage implementation in LMIC settings are reported in the literature. Our analysis provides a comprehensive framework for emergency care leaders planning to implement or scale triage implementation in LMICs.

## Introduction

Emergency care systems (ECS) provide an integrated mechanism to address time-sensitive conditions and an interface to link undifferentiated patients to definitive care. In Low and Middle-Income Countries (LMICs), more than 50% of mortality and one third of disabilities associated with a range of medical and surgical conditions may be prevented by a well-established ECS [1]. The World Health Organization (WHO) has developed a framework that defines the essential ECS functions comprising prehospital care and transport through emergency unit care to early operative and critical care. These functions translate into a package of essential emergency care services, which includes facility-based triage [2].

As LMICs carry a significant burden of the morbidity and mortality associated with many emergency conditions, it is imperative that emergency care provision is strong. With currently limited access to emergency care, strengthening emergency care facilities is paramount. Thus, triage, as part of emergency-based care, represents a major priority need [3].

To this end, a number of triage tools have been developed for low resource settings, including the South African Triage Scale (SATS), Emergency Triage Assessment and Treatment (ETAT), and Interagency Integrated Triage Tool (IITT) [4–6]. These tools have shown acceptability, reliability, and validity in variable facility-based settings [7–17]. Nevertheless, there are continued efforts to develop triage tools that can be feasibility implemented in other LMICs [3].

A number of barriers and facilitators have been reported in the literature for the implementation of these triage tools. These barriers included challenges relating to lack of legislations and standardized protocols, staff training on triage application and awareness on the importance of triage, infrastructure and patient flow, overcrowding and access block, high workload and high staff turnover, resources availability, and difficulties in data entry in new registries [18–23]. To our knowledge, these barriers and facilitators have not yet been consolidated in a systematic way to aid in future triage implementation efforts.

The Consolidated Framework for Implementation Research (CFIR) was designed to consolidate constructs from multiple implementation theories into a single, comprehensive framework to guide systematic assessment of implementation of interventions [24,25]. CFIR has been widely used in global health and implementation science to evaluate both barriers and facilitators across diverse settings, including low- and middle-income countries [26,27]. The framework has been updated in 2022 [25]; it comprises five major domains: (1) Innovation, which assesses the attributes of the “thing” being implemented; (2) Outer Setting, which considers the external environment including patient needs, resources, and policies; (3) Inner Setting, which focuses on the structural, cultural, and resource context within organizations; (4) Characteristics of Individuals, which captures the knowledge, beliefs, and self-efficacy of those involved in implementation; and (5) Process, which examines the planning, engaging, executing, and reflecting activities that drive implementation [24,25].

This framework provides a structured lens for analyzing barriers and facilitators’ implementation of various interventions. By mapping findings to CFIR, this review identifies the factors that shape the implementation of triage practices in healthcare settings across LMICs with the ultimate goal of providing guidance to facilities in resource-limited contexts that plan to implement triage.

Providing a framework of these barriers will help facilities incorporate their assessment and the plan to overcome them into the implementation plan. Therefore, the purpose of this systematic review is to identify the barriers and facilitators to facility-based Triage implementation that are published in the peer-reviewed literature and map them to the Consolidated Framework for Implementation Research (CFIR) to provide a list of these barriers organized within the CFIR domains.

## Methods

Given the lack of review previously conducted on barriers and facilitators to triage implementation, we conducted a scoping review, rather than a systematic review, to capture the scope and breadth of available evidence.

The scoping review was conducted following the Joanna Briggs Institute (JBI) methodology for scoping reviews [28] and reported in accordance with the Preferred Reporting Items for Systematic Reviews and Meta-Analyses extension for Scoping Reviews (PRISMA-ScR) guidelines [29]. A completed PRISMA-ScR checklist is provided in S1 Checklist.

A protocol for this scoping review was prospectively registered in PROSPERO (CRD42024611079).

### Eligibility criteria

The SPIDER framework was used to develop eligibility criteria for this review [30].

For this review, triage was defined as the process of sorting patients presenting for unscheduled or urgent care at any facility-based entry point, including emergency departments, primary healthcare centers, and obstetric or labor and delivery units, to prioritize care according to clinical urgency. This review focused on facility-based triage; prehospital and community-based triage were considered outside the scope of this review.

**S = Sample**

- Healthcare workers, including physicians, nurses, nurse practitioners, emergency medicine technicians
- Policy makers, hospital administrators
- Any type of healthcare facility, that provides emergency or unscheduled care, including primary healthcare centers, emergency departments, labor and delivery floor
- Participants must be based in a low- or middle-income country as categorized by the World Bank

**PI = Phenomenon of interest**

- Research related to barriers and facilitators to triage implementation
- Patient perspectives on triage were excluded if they did not pertain to barriers or facilitators of triage implementation
- Protocol descriptions for triage implementation were excluded

**D = Design**

- Research methodologies including prospective or retrospective cohort studies, case studies, interviews, cross-sectional surveys, implementation studies
- Systematic and scoping reviews were excluded

**E = Evaluation**

- Studies that present findings on barriers and facilitators to triage implementation

**R = Research type**

- Any study design including quantitative, qualitative, and mixed method
- Interventional studies were included
- Grey literatures were excluded
- Studies published in English were included

### Search strategy

A rigorous search strategy was employed to identify all peer-reviewed studies that reported barriers or facilitators of triage implementation in LMICs. We performed a scoping review using the following databases: Scopus, Web of Science-Core Collection, Ovid MEDLINE, Embase, Cochrane CENTRAL, CINAHL, AfricaWide, and Global Health.

The initial search strategy (S1 Text) was developed within Scopus and adapted for the remaining databases. Search terms included various iterations of triage, emergency care, implementation barriers and facilitators, and LMICs. Free-text terms and standardized MeSH headings/ controlled vocabulary were used to optimize sensitivity for relevant literature while minimizing excess search results. The strategy was developed in collaboration with a medical librarian (AB), who also submitted it for peer review within the library’s internal quality assurance process using the PRESS form [31]. The finalized strategy was then shared with the research team to confirm its inclusiveness and accuracy before conducting the full search. The reference lists of relevant primary studies and reviews likely to meet inclusion criteria were also reviewed manually to both verify search sensitivity and identify other potentially relevant studies that were not identified by the electronic search. The search was performed in October 2025.

### Study selection

Manuscripts meeting initial broad search criteria were imported into Covidence (Covidence systematic review software, Veritas Health Innovation, Melbourne, Australia) and duplicates removed. Initial title and abstract review were performed by two independent authors (GE and SA). Disagreements were resolved by a third reviewer (HM). The same procedure was followed for full-text review.

### Data extraction and charting

A structured data extraction tool was developed in Covidence, incorporating a list of predefined themes for barriers and facilitators of Triage implementation. This list was developed deductively by one reviewer (GE), drawing from the literature and refined through discussion with the other reviewers (SA and HM). Barriers and facilitators were extracted from the results and discussion sections of each included study and mapped to one or more of these predefined themes.

Variables extracted included:

- **Publication details**: title, author, year of publication
- **Study details**: design, facility type, healthcare provider characteristics (if applicable), triage tool (if applicable), data collection period, country/region where study was conducted
- **Key findings**: barriers and facilitators to triage implementation

The articles were distributed between two reviewers (GE and SA). Data from each included study were extracted by one reviewer and independently verified by the second reviewer, with any discrepancies resolved through discussion and consensus.

A pre-defined list of barriers and facilitators was initially developed deductively by one reviewer (GE), drawing on triage and implementation-science literature published prior to the start of this review, and refined through discussion with the other reviewers (SA, HM). During data extraction, this list was refined inductively using the included studies: barriers and facilitators that did not align with an existing theme were temporarily coded as “other” by the extracting reviewers (GE, SA), then reviewed collaboratively, with themes added, merged, or subdivided to accommodate emerging concepts and any disagreements resolved through discussion and consensus with the third reviewer (HM). This list can be found in S1 Table.

### Quality appraisal

We used JBI critical appraisal tools to assess the methodological quality of included sources based on relevant study type [32]. Mixed method studies were assessed using the Mixed Methods Appraisal Tool (MMAT) [33]. Studies were evaluated into trichotomous ratings (low, medium and high risk of bias) using the criteria below:

- Low: All or almost all of the criteria were fulfilled, and those that were not fulfilled were thought unlikely to alter the conclusions of the study
- Medium: Some of the criteria were fulfilled, and those not fulfilled were thought unlikely to alter the conclusions of the study
- High: Few or no criteria were fulfilled, and those that were not fulfilled were thought likely or very likely to alter their conclusions

No studies were excluded based on quality, in line with scoping review guidance.

### Evidence synthesis

We conducted a qualitative synthesis of the included studies to identify reported barriers and facilitators to triage implementation. For each barrier and facilitator, operational definitions were developed and refined to clarify the constructs being captured and to promote consistency in coding. Once the final list of barriers and facilitators was established, two authors (GE and SA) independently mapped it to the domains of the CFIR, and the third reviewer (HM) reviewed and confirmed the reconciled version. The domains of the CFIR framework were adapted to triage for the purposes of this review. The original and adapted definitions are detailed below:

- **Domain 1:** Innovation
  - *CFIR definition:* Attributes of the intervention, including its evidence strength, adaptability, trialability, complexity, and cost.
  - *Application to triage:* The degree to which triage protocols are evidence-based, adaptable to local contexts, easy to use and feasible to implement in resource-constrained settings. It also includes implementation costs.
- **Domain 2**: Outer Setting
  - *CFIR definition:* The external environment influencing implementation, such as patient needs and resources, peer pressure, and external policies or incentives.
  - *Application to triage*: The role of person/community expectations, healthcare system funding, national health policies and laws, international guidelines, integrated linkages with prehospital and primary care systems, and the impact of large-scale or unanticipated events that may disrupt implementation or delivery of the innovation in supporting or hindering triage uptake.
- **Domain 3**: Inner Setting
  - *CFIR definition*: The structural, cultural, and resource context within implementing organizations.
  - *Application to triage*: Organizational mission and culture, internal communication, resource availability, hospital policies and processes, the security of the environment, and the layout and infrastructure of the hospital and department.
- **Domain 4**: Characteristics of Individuals
  - *CFIR definition:* The knowledge, beliefs, self-efficacy, and individual readiness of those involved in implementation.
  - *Application to triage*: Providers’ competence level and attitudes toward triage, confidence in using tools, perceptions of their role within triage, the contributions of all members of the implementation teams, and the leadership support in enabling effective implementation.
- **Domain 5:** Process
  - *CFIR definition:* The specific implementation processes of planning, engaging, executing, and evaluating.
  - *Application to triage:* How triage is introduced through structured planning and implementation, champions are engaged, staff are trained, documentation is maintained, and feedback loops are established to adapt and sustain practice.

Using this approach, the barriers and facilitators identified across the included studies were grouped into five CFIR-aligned classes of determinants for triage implementation. Data was narratively synthesized, with specific findings (including qualitative quotes) extracted where suitable.

## Results

A total of 1,586 articles were identified, with 57 articles included after screening (Fig 1). Detailed table of study characteristics is located in S2 Table. Barriers and facilitators were identified across all five CFIR domains. The 57 studies represented findings from 43 countries. Of these, 30 (52.6%) were quantitative, 12 (21.1%) were qualitative, 11 (2.2%) were mixed methods, and 4 (7%) were editorials.

**Fig 1:**
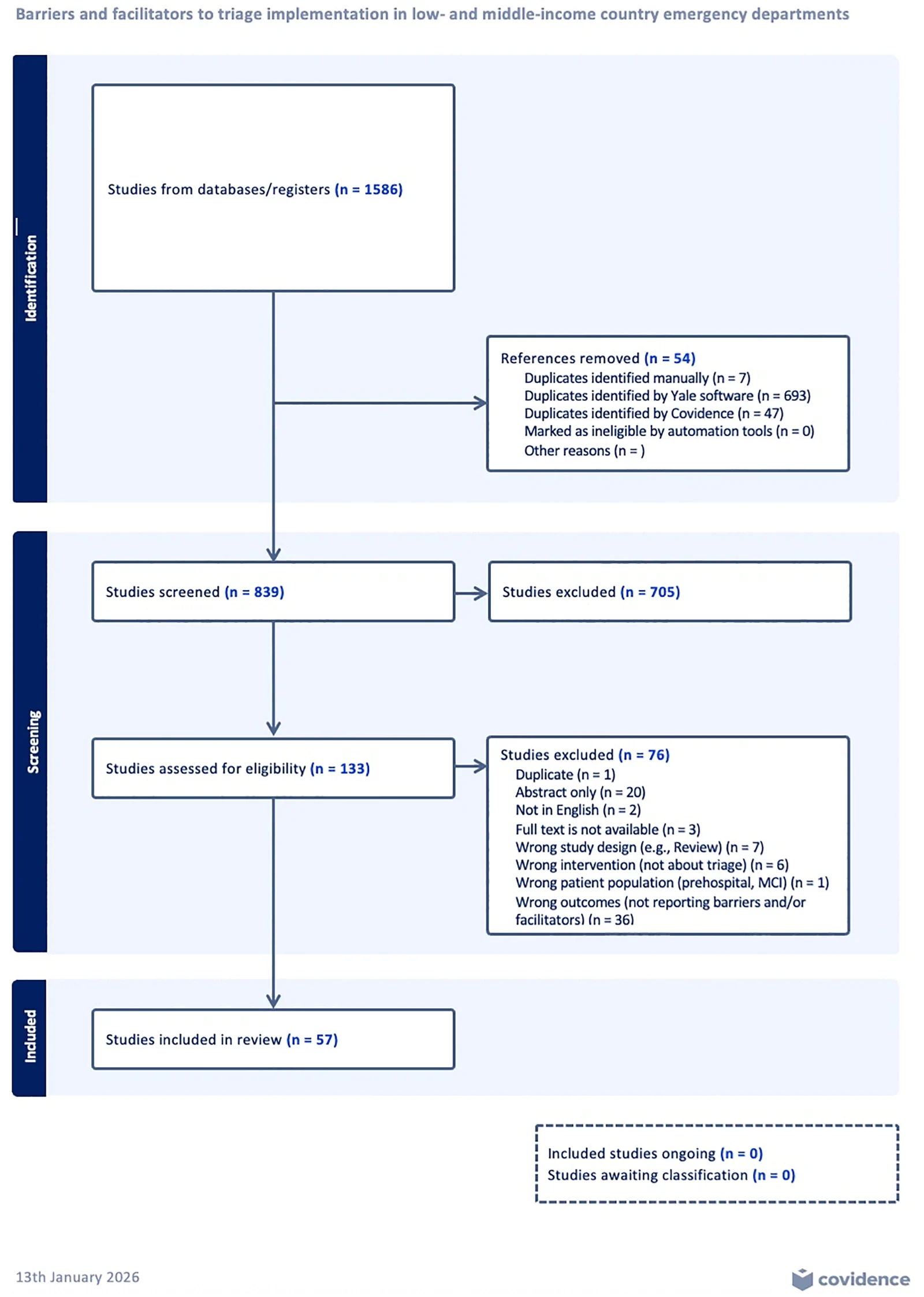
PRISMA flow diagram of the scoping review study selection process.

Geographically, publications included data from 6 WHO regions, with the majority from the Africa Region 26 (47.3%) and all were from lower or middle-income countries by World Bank grouping (2025). Two articles (3.6%) included data from multiple countries spanning income groups (Table 1).

**Table 1:** Characteristics of included studies.

Characteristic	Studies, n(%)	Countries, n
WHO Region		
Africa	26 (47.3%)	14
Americas	8 (14.5%)	6
Eastern Mediterranean	9 (16.4%)	4
European	1 (1.8%)	1
South-East Asia	6 (10.9%)	3
Western Pacific	8 (14.5%)	8
Multiple WHO regions	2 (3.6%)	_
Income Level		
Low-Income	15 (27.3%)	7
Lower-Middle	14 (25.5%)	9
Upper-Middle	19 (34.5%)	8
Multiple Income Categories	2 (3.6%)	_
Undefined	2 (3.6%)	2
Study Type		
Quantitative	30 (52.6%)	18
Qualitative	12 (21.1%)	20
Mixed	11 (19.2%)	12
Editorial	4 (7%)	4
Barriers/Facilitators as outcomes		
Yes	47	30
No	10	6

### Quality appraisal

All 57 studies underwent critical appraisal using JBI tools or MMAT for mixed methods studies. Of these, 37 (65.0%) were categorized as low risk of bias and 20 (35.0%) were categorized as medium risk of bias. Appraisals of each study are detailed in S3 Table. The most common reason for medium risk of bias was lack of consideration of confounders in the cross-sectional studies.

### Evidence synthesis

Facilitators were most frequently reported, with a predominance noted in Domain 5 (n = 47). There was some overlap (i.e., some articles discussed more than one domain or both barriers and facilitators). A summary of the literature by CFIR domain are displayed in Fig 2.

**Fig 2:**
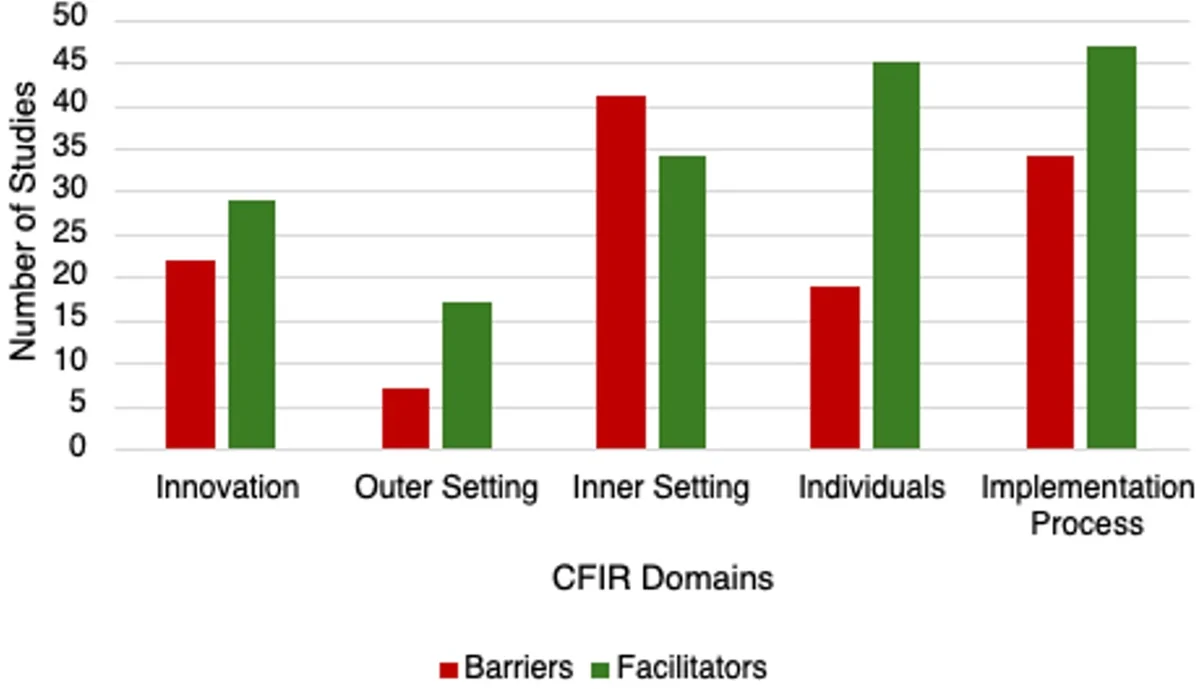
Identified barriers and facilitators to triage implementation from the literature–categorized by the consolidated framework for implementation research as a guide.

#### DOMAIN 1: Innovation.

42 studies discussed Domain 1 [18,19,21,23,34–71] with 22 (38.6%) studies discussing barriers [18,19,21,23,34,36–38,41,42,47,51,52,54,58,61,65–68,70,71] and 29 (50.9%) discussing facilitators within this domain [18,34,35,37–40,43–46,48–51,53–57,59–65,68,69].

##### Barriers.

Many of the interventions were noted to not be adaptable either to local contexts or specific populations. This was seen in the implementation of an obstetric triage tool in Iran—one provider noted:

*“It appears that only older pregnancies are considered, while there are very critical early pregnancies, such as ectopic pregnancy, which should also be respected”(M14)* [41]

Furthermore, if triage was complex to perform, this was noted to be a major barrier—in Ghana, 87.28% of healthcare providers surveyed noted that this was within the top 3 major barriers to performing pediatric triage [47].

##### Facilitators.

Many studies noted that the triage tool was seen effective in differentiating between sick and non-sick patients. A healthcare provider in Belize, where ETAT was implemented noted that:

*“The ETAT helped a lot so that we could identify, differentiate between a real sick child compared to a child who could wait longer”* [61].

Simpler triage systems that are easily adaptable, and which are based on clinical symptoms, were perceived to be more suitable for resource-limited settings. In a study implementing IITT in a resource-limited hospital in Nepal, several participants reported that the three-tier, symptom-based system was easier to use than the four-tier Rapid Emergency Triage and Treatment System (RETTS) model, which they felt was too complex for day-to-day operations in the Dhulikhel Hospital Emergency Department (DHED). They appreciated the focus on clinical presentation rather than diagnostic testing, noting that access to tests was often limited by cost, infrastructure, and available technology [68].

Coding systems within triage tools were reported as facilitators to implementation. In Uganda, where Smart Triage was implemented, a clinical officer noted:

*“It has helped in a way that if I see a child putting on a red lanyard, even if I have someone there with yellow, I will have to tell them, “First order, because I need to send this one as fast as possible, you know. So it has helped me to attend to emergencies better as compared to before””[Participant 12, OPD]* [60].

Similar findings were reported in Uganda where 100% of staff strongly agreed or agreed that creating and labeling areas of triage categories would improve triage (Opiro 2017). [21]

In Zambia, 70.0% of staff reported that having a triage area and system has improved the organization of their units [72].

#### DOMAIN 2: Outer setting.

19 studies discussed Domain 2 [18,19,21,23,35,37–39,41,43,46,49,54,59,60,64,65,73,74], with 7 (12.3%) studies discussing barriers [23,37,38,41,43,46,64] and 17 (29.8%) discussing facilitators within this domain [18,19,21,35,37–39,43,46,49,54,59,60,64,65,73,74].

##### Barriers.

Conflicting protocols were noted to be a barrier to implementation. In Rwanda, a trainee attending ETAT+ training noted that there were varied guidance between national, hospital, and ETAT+ protocols:

*“There are a lot of guidelines out there, don’t know which one to use or not, particularly in neonatology”* [18].

This was also noted as a barrier in Uganda, where 100% of participants surveyed noted that a major barrier to implementation was a lack of a national triage system, and that a unified national triage approach was needed [21].

##### Facilitators.

In Uganda, 100% of staff across 7 facilities strongly agreed that adopting a unified nationwide triage scale would improve triage [21]. In Cuba, the integration of the public health system as well as the development of nationwide primary care training was also noted to be a facilitator [35].

In Malawi, there was high levels of engagement with the Ministry of Health which facilitated implementation of ETAT in primary care settings [64].

#### DOMAIN 3: Inner setting.

50 studies discussed Domain 3 [18–21,23,34–43,45–49,51–71,73–81] with 41 (71.9%) studies discussing barriers [18–21,23,34,36–43,46–48,51–53,55–63,65–68,70,71,74–76,78–80] and 34 (59.7%) discussing facilitators [19,21,34–37,39,40,45,46,48,49,51,53–57,59–65,68,69,73–75,77,79–81] within this domain.

##### Barriers.

Lack of equipment was cited throughout the literature as a significant barrier to triage implementation as well as the downstream processes of triage [18,19,23,34,36–38,40,42,47,48,51,53,55–63,65–68,70,74,75,79,80]. One participant from Madagascar where ETAT+ was implemented noted:

*“…the resuscitation phase requires the use of oxygen... but in the case of our institution, we have no source of oxygen... only the CHU of Tuléar around here has a working oxygen extractor... The training is undoubtedly feasible and relatively simple even... but it is the practical implementation, which is problematic...” (Interview no. 2 en: 22–22)* [36].

Lack of triage equipment was noted In Belize, where ESI was implemented, healthcare workers reported:

*“We’ve made it a habit of everyone having their own equipment”* [61].

Despite the culture of healthcare providers having their own equipment in Belize, another provider noted:

*“Not all of the nurses can afford to buy their own equipment. Some of us, our lifestyles are different, and we have financial difficulties, so I believe that if you are working at a facility, that facility should provide you with the appropriate equipment”* [61].

In Malawi, the availability of functional equipment required for ETAT in Malawi ranged between 13–81% [23]. There were also some instances in which equipment was available but could not be used. One healthcare worker noted:

*“In our case we have a glucometer which we can use, but when we went to the [District Health Office] to order the strips, we were told they don’t match. As a result, it is just as good to say we don’t have the glucometer” (Zomba, FGD 3)* [23].

There was also varied availability of essential drugs ranging between 16–100% [23].

In some contexts, physical infrastructure of clinical areas as well as staff shortages and patient flow were found to be significant barriers to triage implementation. A clinician in Papa New Guinea noted:

*“Triaging system is great however we have a very limited space as well as staff and the patient overflowing is too overwhelming”* [20].

In South Africa, where South African Triage Scale (SATS) was implemented, 59% of respondents to a survey agreed or strongly agreed that lack of human resources posed a hindrance to the use of triage. Further, 51% of respondents agreed or strongly agreed that overcrowding in waiting rooms was also a barrier to the use of triage [66].

Another barrier was the work schedules of trainees; in Rwanda, on-site ETAT+ trainings had high dropout rates due to staff not being given protected time to attend. One trainee noted:

*“I attended the training held in my hospital, and had been on night call every other day because of limited staff in my department”* [18].

Another clinician in Zambia noted that the community also needed to be engaged and aware of triage procedures for its success.

*“We need to teach people about triage, how we are supposed to do it and what we can do at the hospital…. sensitization on triage should be done so that people can be aware that once there is an emergency we will first attend to that emergency”* [72].

While the use of triage is critical, it functions within a broader system of emergency care, and its effectiveness depends on clear direction for patient management after a triage level is assigned. In the study adapting IITT tin Nepal, participants noted that, although there was interest in adopting the IITT, successful implementation depended on having explicit guidance on the actions required once a triage category was determined. As one participant explained,

*“The problem is not, ‘What is wrong with the patient?’ It is ‘What do I do next?’”* [68].

Security factors were also identified as potential barriers to triage implementation. In the study adapting IITT in Nepal, nurses reported concerns about personal safety, describing situations in which they felt threatened by patients or visitors when enforcing triage decisions [68].

##### Facilitators.

Effective referral and communication pathways were noted to be facilitators of triage implementation. During the COVID pandemic, healthcare facilities in the Pacific regions had referral patterns in place, making it easy to implement triaging and screening in their emergency care pathways. One clinician noted:

*“Before they send or refer a case whoever is referring, [we’ll] update the ED first, ‘Look we're sending this case over to you guys and this is what we [referral hospital] have done’… We're hoping with the establishment of the ambulance and the emergency centre in [our country] [they can] help us solve the burden of work especially in terms of choosing which patients [are or aren't] supposed to be referred to [the] ED”* [43].

On-site mentorship was a cited frequently as a facilitator to implementation. Notably, in Iran where obstetric triage was implemented, one policymaker noted:

*“Experienced midwives help with the first-year resident that the patient is not neglected. Through their expertise, they comprehend something that the novice resident does not recognize”(P4)* [41].

#### DOMAIN 4: Characteristics of individuals.

51 studies discussed Domain 4, [18,19,21,23,34–41,43–46,48–66,68–71,73–77,79–85] with 19 (33.3%) studies discussing barriers [18,23,36–38,41,43,51,52,55,57,58,60,62,65,66,70,76,80] and 45 (79.0%) discussing facilitators [18,19,21,23,34–41,44–46,48–51,53–57,59–65,68,69,71,73–75,77,79–85] within this domain.

##### Barriers.

A notable barrier was the reluctance of some individuals to change. A healthcare worker in Rwanda noted:

*“Old people, senior staff…particularly MDs [medical doctors] if not trained don’t comply with new guidelines compared to nurses”* [18].

Lack of provider knowledge was also cited as a barrier to implementation of triage; in Iran when implementing a triage tool for obstetrics, one clinician noted:

*“Sometimes resident one year had discharged the patient that had urgency symptoms because she is not yet completely aware of the problems and complications of pregnancy”(P2)* [41].

In this same context, some providers did not see the point of doing triage, with one policymaker noting:

*“Some people oppose it and say there is no need to perform OB triage, we would have done our duty without it.”(P5)* [41].

This was seen also notably in South Africa where 54% of respondents to a survey agreed or strongly agreed that nurses waiting for doctors when they already know what to do was a barrier to using the SATS [66]. Lack of patient orientation to the triage process was also identified as a barrier. In Nepal, one participant reflected:

*“I didn’t know about triage and had never noticed it in other hospitals before. Now I understand it. If I get the chance, I’ll explain it to others, and I’ll remember it if I come across it in other hospitals”* [65].

Lack of support from hospital leadership was also noted to be a barrier in some settings in the Pacific region, with one clinician noting:

*“The biggest challenge is trying to set up the SCOVID screening area in front of the ED. It hasn't happened. We tried to meet with the executive, our leaders, but still now we're unable to establish it…At the moment we don't have any case[s], and we're still trying to set up the ED SCOVID screening area”* [43].

##### Facilitators.

The literature highlights the perceived usefulness and value of triage systems among emergency care providers as facilitators of their implementation across diverse emergency care settings.

In a study implementing IITT in PNG, there was 100% agreement that *“the new triage and flow system helps identify and prioritise the most urgent patients”* [20].

In another study adapting IITT to a resource-limited hospital in Nepal, using a 5-point Likert scale ranging from “Not at all important” to “Very important,” all respondents rated triage as “Very important.” Participants described triage as a critical process for ensuring timely treatment of critically ill patients and doctors’ prioritization of resources, space, and attention [68].

*“Without triage, there is chaos [in the ED]”* (Weiner 2025) [68].

#### DOMAIN 5: Implementation process.

53 studies discussed Domain 5 [18–21,23,34–37, 39–69, 72–77,79–85] with 34 (59.65%) studies discussing barriers [18–21,23,36,37,39–42,47,48,52,53,56–59,61–63,65,66,68,72–76,79,80,82,85] and 47 (82.46%) discussing facilitators [18,19,21,23,34–37,39–41,43–46,48–65,67–69,72–75,77,79–84] within this domain.

##### Barriers.

Language was noted to be a barrier within training or training materials in several studies [REF}. In Rwanda, one trainee noted:

*“Training, and booklet [training materials] are in English that I barely understand”* [18].

With regards to training methods, although web-based training was found to be theoretically acceptable, for some this was a barrier in Papa New Guinea where one trainee mentioned:

*“The training itself was excellent, the only problem was data to go online”* [63].

In South Africa, 84% of respondents to a survey agreed or strongly agreed that lack of adequate funding for nurses to attend international workshops was a barrier to using SATS [66].

##### Facilitators.

Several facilitators within Domain 5 were cited with regards to implementation of triage. A study conducted in the Hunan province of China found that 75.4% of nurses preferred to develop triage skills through a mentor or more experienced nurse, 64.9% preferred books or other printed material, and 53.8% of nurses preferred conferences [82]. Many of these factors are noted to be facilitators cited throughout the literature in our scoping review as facilitators in various settings. For example, in Rwanda, where ETAT was implemented, trainees found the booklet they were provided to be helpful in implementing ETAT. One trainee reported:

*“Using the while booklet [Rwanda basic pediatrics protocols] as we were auditing medical record helped integrating [ETAT+] materials”* [18].

Still, there were other factors within this domain that were found to be facilitators, such as hospital audits. This was also done in Rwanda, and one trainee noted:

*“I found hospital audit helpful and engaging, as I could see what was done wrong, and discussed with others what could have been done based on [ETAT+ clinical practice guidelines] recommendations”* [18].

Regular training was also noted to be a potential facilitator to the use of triage—in Uganda, 100% of staff strongly agreed that regular training would improve triage in hospitals [21]. 100% of staff also strongly agreed that printing the triage tools and putting them in open places in emergency receiving areas would improve triage practices [21]. This was corroborated by another study conducted in Nepal, where 45.6% of healthcare workers perceived the need to improve triage training and supervision among staff [65]. In Bolivia, after a 4-week training course, knowledge to apply the Manchester Triage Score increased significantly in comparison to their baseline (*p < 0.05*) [29].

## Discussion

This is the first scoping review, to our knowledge, that maps barriers and facilitators of triage implementation in LMIC settings within the five domains within the CFIR framework. LMIC countries span many continents and different geopolitical contexts, but many of the barriers and facilitators are noted to be similar across and within each of these countries.

A majority of our studies were of low risk of bias, indicating that while triage implementation is not a major area of research, the research that has been done has been rigorously done. There is also a wide array of methodologies employed in the study, although about half of the studies were quantitative in nature.

Much of the evidence echoes what is noted in high income settings. For example, in Norway, managerial support as well as national policies that supported triage were noted to be significant facilitators to triage implementation [86]. Nevertheless, there are many barriers and facilitators that may be more frequently experienced in LMICs, such as equipment shortages.

Our review reveals gaps in research across different regions within LMICs. While Sub-Saharan Africa represents many countries within this category, many of the studies were done in a limited number of countries. This clustering may reflect the presence of established research partnerships, donor priorities, or regional centers of excellence in emergency and acute care research. Other regions, such as the Americas, the Eastern Mediterranean regions, and Southeast Asia, the Western Pacific, and Europe were underrepresented, ranging between 10–17% of the total literature included within the review. This may have implications for the transferability of findings, as implementation barriers and facilitators are highly contextual. Findings from limited geographic areas may not reflect realities in the other underrepresented regions with different system structures, staffing, financial models, and policy environments.

Future research is recommended to examine implementation facilitators and barriers in underrepresented regions, and to intentionally assess how contextual factors vary across settings, including via multi-country or cross-regional comparisons, to strengthen generalizability and guide adaptation of triage interventions to diverse system and facility contexts.

Overall, facilitators were more frequently identified than barriers in most areas. However, facilitators were not overrepresented. Thus, the available research offers balanced reporting of barriers and facilitators, with a slight emphasis on practical strategies that support triage implementation. The largest number of facilitators were reported in the Individuals and Implementation Process domains, suggesting that workforce engagement, training, and implementation strategies were perceived as facilitators for triage adoption. The Inner Setting domain, however, demonstrated a slightly higher number of barriers, highlighting possible organizational and structural challenges within facilities. This also underscores the importance of engaging stakeholders at leadership and decision-making levels. This will help them be prepared for the expected contributions and responsibilities on their end to support the success of implementation. Hospital managers may be more responsive to shared experiences from leaders of facilities with similar settings, exchanging ideas about operational lessons and demonstrated successful strategies, than to attending triage training that is focused on using the tool in clinical practice. Strengthening leadership buy-in and support may present a strategic and pragmatic approach to addressing inner setting barriers.

Outer Setting was the least frequently mentioned domain overall. This might be, in part, because external factors, such as national policies, regulations, and other system-level constraints, are often less amenable to modification by individual clinicians or facilities and may therefore be less emphasized by facility-based studies. Additionally, many included studies were not designed using the CFIR framework and as a result, outer setting factors may not have been identified or discussed in a way that clearly maps them to CFIR constructs. This finding is consistent with some literature, such as a recent scoping review examining telehealth implementation [87] but at odds with others, such as a scoping review looking at healthcare interventions implemented for vulnerable communities [88].

The wide range of identified barriers and facilitators underscores the complexity of triage implementation in LMIC settings and highlights the importance of advanced assessment of contextual barriers and facilitators. Such assessment enables the design of tailored implementation plans that address specific challenges while leveraging existing opportunities to enhance feasibility and sustainability. The factors identified in this review can serve as a practical framework or checklist to be used both in the planning and implementation phases. This may aid implementers to prioritize interventions, allocate resources, and consider the time and effort required to address barriers within their plans. This approach is frequently used in implementation research, where multiple frameworks, strategies, and behavior change methods are employed to enhance efficiency of implementation [89].

## Strengths and limitations

Our review has several strengths and limitations. It is the first scoping review to examine triage implementation in LMICs. The review followed JBI methodology, used the SPIDER framework to curate the search, and was reported in line with PRISMA-ScR guidelines. Quality appraisals were also done using JBI tools and MMAT for mixed methods studies. By applying CFIR, the review provides a structured lens for analyzing barriers and facilitators to triage implementation. Our results may therefore help guide emergency care planners to proactively identify and address barriers and facilitators during the planning phase of triage implementation or improvement, which may facilitate more effective implementation.

Despite the study’s strengths, there are several limitations. In 10 out of 57 studies, barriers and facilitators were described as part of authors’ interpretations within the discussion sections of their studies. Thus, they were not analyzed as formal study outcomes. Additionally, there was limited literature that purposefully explored barriers and facilitators to triage implementation. In fact, many included studies did not apply a formal implementation science framework. Furthermore, grey literature was not included. This introduces publication bias and may have resulted in omission of programmatic or operational insights from non-peer-reviewed sources. A recent scoping review noted that primary data offered through grey literature may be used to facilitate further research in under-researched communities, underscoring its importance especially in LMICs where research infrastructure may still be in its early stages [90].

The heterogeneity of included studies, such as differences in triage tools, patient populations, and facility types, limits the generalizability of these findings across LMIC settings. Finally, most studies focused on initial triage implementation, with little to no attention to its sustainability. Thus, long term triage use cannot be commented on via our review.

## Recommendations

Facilities may apply the identified barriers from this review as a contextual framework or checklist for triage implementation by initially reviewing intervention-related challenges. Fig 3 can be used as a reference checklist. Definitions of each domain and factor, including how factors were operationalized as barriers versus facilitators and illustrative examples, are provided in S4 Table. For example, complexity of triage tools can act as a barrier to implementation. Choosing a tool that has demonstrated success in similar low-resource environments—and piloting it with staff—can support effective adaptation. Engaging key stakeholders during the selection and modification process can enhance both usability and acceptance. It is also important to assess outer setting factors, especially policies surrounding emergency care in the country of implementation. Where triage policies are absent at the national or institutional level, implementers can collaborate with leadership and partners to develop policies that formally integrate triage into emergency care systems.

**Fig 3:**
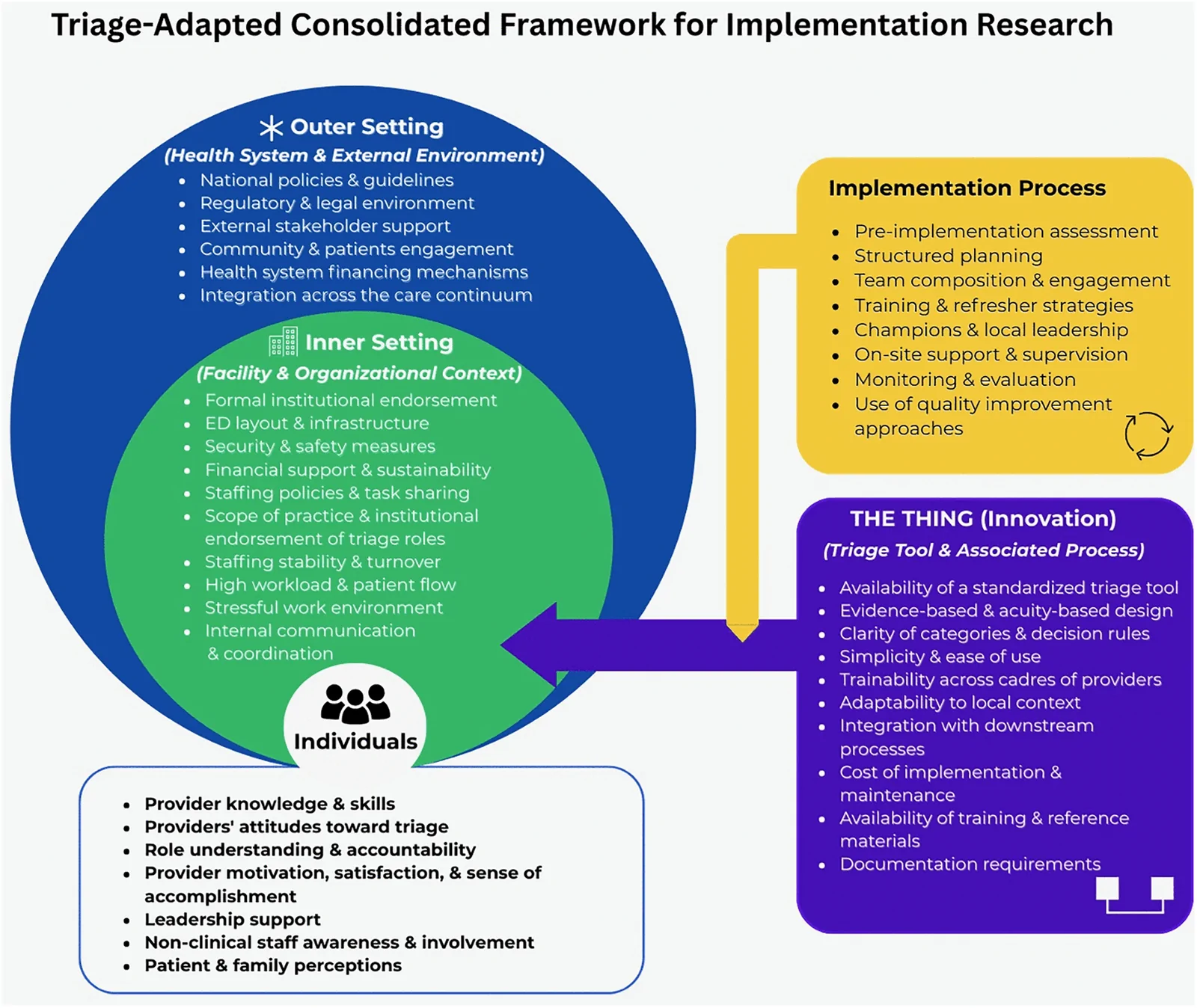
Triage-adapted consolidated framework for implementation research – Adapted from the updated consolidated framework for implementation research based on user feedback [25].

Attention should also be given to inner setting barriers, including limitations in emergency unit infrastructure. An initial action may include mapping the current ED setup, reassigning space to accommodate triage activities, and redesigning patient flow for better efficiency and safety. Regarding the individual characteristics, gaps in staff training may limit effective adoption. To mitigate this, training should be built into the early stages of implementation, with plans and budgeting for continuous and refresher training. Lastly, addressing the implementation process is critical. Baseline assessments of the context and the chosen triage tools should be done to ensure adaptability to available resources. Incorporating a monitoring and evaluation (M&E) component from the beginning is also crucial to enable tracking of implementation progress, early identification of issues, and timely adjustments—improving the likelihood of sustained success.

## Conclusion

Implementation and sustaining facility-based triage in LMICs is a complex and challenging process. Providing a framework or a checklist of potential barriers and facilitators categorized into domains can help emergency department planners identify the specific factors within their own context early in the implementation plan. This will enable them to incorporate implementation strategies tailored to their context, aiming at establishing a triage system that is feasible and sustainable, and improve patient care and outcomes.

## Supporting information

S1 Text Search strategy. Detailed search strategy used for the review.
(PDF)

S1 Table List of predefined themes. List of predefined themes used to categorize barriers and facilitators.
(PDF)

S2 Table Included studies. Characteristics of studies included in the review.
(DOCX)

S3 Table Quality appraisal. Quality appraisal of the included studies.
(DOCX)

S4 Table Domain and factor definitions, operationalization as barriers and facilitators, and illustrative examples based on the Consolidated Framework for Implementation Research (CFIR). (PDF)

S1 Checklist PRISMA 2020 checklist. The PRISMA 2020 checklist is distributed under the Creative Commons Attribution License (CC BY 4.0).
(DOCX)
