# Supplementary material for: Identifying barriers and facilitators to implementing triage systems in low- and middle-income countries: A scoping review
Source: PLOS Glob Public Health. 2026 Sep 29;6(9):e0007378. doi: 10.1371/journal.pgph.0007378 (PMC13623090; doi:10.1371/journal.pgph.0007378)
Supplement: Supplementary file 1 [file pgph.0007378.s001.pdf]

S1 Text. Example Search Strategy

Line	Search terms	Search results
	Ovid MEDLINE(R) ALL <1946 to October 28, 2025>	
	Search terms	Search results
1	emergency medical services/ or emergency service, hospital/ or trauma centers/	156813
2	((emergency or emergent or urgent or casualty) adj2 (department* or service* or care or treatment or center* or area* or ward* or unit* or facilit* or provider* or physician* or clinician* or nurs* or care or staff* or treat*)).mp.	300347
3	1 or 2	311093
4	triage/	16461
5	(triage adj3 (system* or process* or unit* or tool* or score* or scale* or algorithm* or framework* or guideline* or pathway* or criteria* or intervention* or implementation)).mp.	7638
6	4 or 5	20124
7	(implementation* or barrier* or facilitator* or challenge* or obstacle* or constraint* or enabler* or opportunity* or adaptation* or development*).mp.	6391165
8	(implementation science or implementation framework* or dissemination or uptake or scale-up or integration).mp.	953051
9	exp Implementation Science/	1857
10	7 or 8 or 9	7050934
11	Developing Countries/	84728
12	(developing countr* or low income countr* or middle income countr* or LMIC* or resource-limited or underserved or "Global South").mp.	241983
13	(low adj2 middle adj2 income adj2 countr*).mp.	41338
14	(low-resource countr$ or low-resource setting$).mp.	10922
15	(lmic or lmics or lami countr* or lic or lics).ti,ab.	17192
17	(Afghanistan or "Burkina Faso" or Burundi or "Central African Republic" or Chad or Congo or Eritrea or Ethiopia or Gambia or Guinea-Bissau or Korea or Liberia or Madagascar or Malawi or Mali or Mozambique or Niger or Rwanda or "Sierra Leone" or Somalia or "South Sudan" or Sudan or Syria* or Togo or Uganda or Yemen or Angola or Bangladesh or Benin or Bhutan or Bolivia or "Cabo Verde" or Cambodia or Cameroon or Comoros or Congo or "Cote d'Ivoire" or Djibouti or Egypt or Eswatini or Ghana or Guinea or Haiti or Honduras).mp.	579984
18	(India or Jordan or Kenya or Kiribati or "Kyrgyz Republic" or Lao PDR or Lebanon or Lesotho or Mauritania or Micronesia or Morocco or Myanmar or Nepal or Nicaragua or Nigeria or Pakistan or "Papua New Guinea" or Philippines or Samoa or "Sao Tome" or Senegal or "Solomon Islands" or "Sri Lanka" or Tajikistan or Tanzania or Timor-Leste or Tunisia or Uzbekistan or Vanuatu or Vietnam or "West Bank" or Gaza or Zambia or Zimbabwe or Albania or Algeria or Argentina or Armenia or Azerbaijan or Belarus or Belize or Bosnia or Herzegovina or Botswana or Brazil or China or Colombia or "Costa Rica" or Cuba or Dominica or "Dominican Republic" or Ecuador or "El Salvador").mp.	1238214

19	(Equatorial Guinea or Fiji or Gabon or Georgia or Grenada or Guatemala or Indonesia or Iran or Iraq or Jamaica or Kazakhstan or Kosovo or Libya or Malaysia or Maldives or Marshall Islands or Mauritius or Mexico or Moldova or Mongolia or Montenegro or Namibia or North Macedonia or Paraguay or Peru or Serbia or South Africa or "St. Lucia" or "St. Vincent" or Grenadines or Suriname or Thailand or Tonga or Turkiye or Turkmenistan or Tuvalu or Ukraine or Venezuela).mp.	466839
20	11 or 12 or 13 or 14 or 15 or 17 or 18 or 19	2292549
21	3 and 6 and 10 and 20	291
22	limit 21 to yr="2024 - 2025"	57
