# Supplementary material for: Identifying barriers and facilitators to implementing triage systems in low- and middle-income countries: A scoping review
Source: PLOS Glob Public Health. 2026 Sep 29;6(9):e0007378. doi: 10.1371/journal.pgph.0007378 (PMC13623090; doi:10.1371/journal.pgph.0007378)
Supplement: Supplementary file 2 [file pgph.0007378.s002.pdf]

S1 Table. Final List of Predefined Themes of Barriers and Facilitators of Triage Implementation

Innovation	Facilitators	**Simple and user-friendly tools**	Captures facilitators related to the design and usability of the triage tool itself. This includes aspects such as the tool being easy to understand and quick to learn. Facilitators describing triage systems with clear criteria, minimal complexity, and intuitive formats that enable consistent and efficient use by staff fall under this theme. Easy or straightforward application within the implementation context is captured under the “Contextual and cultural adaptability” theme.
		**Contextual and cultural adaptability**	Captures facilitators related to the flexibility and relevance of the triage tool or system across different settings. This includes its ability to be adapted to the local context, health system structure, and resource availability, as well as evidence of effectiveness in similar environments. Facilitators describing cultural or contextual tailoring—such as language adaptation, alignment with local norms, or modification of procedures to fit existing workflows—also fall under this theme.
		**Visual triage aids**	Captures facilitators related to the use of visual tools or color-coded systems that support effective triage implementation. This includes the application of color strips, tags, or other visual markers to categorize patient urgency, enhance clarity, and streamline decision-making. Facilitators describing visual aids that improve the speed, accuracy, and consistency of triage assessments fall under this theme.
	Barriers	**Complex, deficient and unclear triage tools and protocols**	Captures barriers related to triage tools that are difficult to understand, interpret, or use efficiently. This includes tools with unclear categories or descriptions, excessive complexity, or designs that are time-consuming to apply in practice. Barriers describing deficient or poorly defined protocols, lack of visible guidance, or inadequate signage that contribute to confusion and inconsistent application of triage also fall under this theme.
		**Poor contextualization**	Captures barriers related to the limited adaptability or flexibility of triage tools and systems to different settings and contexts. This includes tools that cannot be tailored to local health system structures, resource levels, or population needs, resulting in poor applicability and reduced effectiveness. Barriers describing difficulties in implementing standardized tools due to the inability to modify or localize them for specific contexts fall under this theme.
Outer Setting	Facilitators	**National legislations and regulatory framework**	Captures facilitators related to the availability of national or regional policies and plans that prioritize emergency care or triage, and national legislation or mandates for a standardized triage system.
		**Effective referral and transport**	Captures facilitators related to the existence of organized and reliable systems for referral, counter-referral, and patient transport. This includes established communication and coordination systems and protocols between facilities, clear interfacility transfer processes, and functional ambulance or prehospital systems that ensure timely and appropriate patient movement. Facilitators describing well-integrated referral pathways and transport networks that support continuity of care and efficient triage fall under this theme.
		**Community engagement and awareness**	Captures facilitators related to the involvement, understanding, and acceptance of triage within the community. This includes existing community awareness of triage processes as well as activities conducted to inform, educate, and sensitize the public about the purpose and importance of triage. Facilitators describing efforts to build trust, improve communication, and foster community cooperation with triage systems fall under this theme.
		**Broader health system**	Captures facilitators related to the alignment and coordination of triage implementation within the broader health system, beyond referral pathways. This includes efforts to strengthen primary care capacity. Facilitators related to effective referral systems to link between the different health system components are captured seprately under "Effective referral and transport".
	Barriers	**Poor System Integration / Fragmented System (Across Levels of Care)**	Captures barriers related to weak coordination and linkages across different levels of the health system. This includes inadequate referral and counter-referral mechanisms and weak continuity and integration between primary care, emergency, and higher-level facilities. Barriers describing fragmentation or inefficiencies in system components, including the prehospital system, that disrupt continuity of care and overload triage services at the broader system level fall under this theme.
		**Institutional and Hospital Policies**	Captures facilitators related to the presence of hospital-level policies and standard operating procedures (SOPs) that formalize and sustain triage practices within facilities.
		**Integrated teamwork practice**	Captures facilitators related to effective collaboration and coordination among healthcare workers and departments involved in triage and emergency care. This includes multidisciplinary teamwork, clear communication, and professional cooperation within and across departments. Facilitators describing strong interdepartmental coordination, shared responsibility, and integration of roles to ensure efficient and seamless triage processes fall under this theme.
		**Efficient layout and infrastructure**	Captures facilitators related to the organization, design, and adequacy of the physical environment supporting triage. This includes the availability of sufficient and appropriately located triage stations, dedicated emergency and resuscitation areas, and clearly assigned spaces for different triage categories. Facilitators describing improved spatial layout, patient flow pathways, waiting areas, and physical arrangements that enhance privacy, visibility, and efficiency of movement through the triage process fall under this theme. Availability of transfer trolleys and essential contact information within the triage area is also captured under this theme.

Inner Setting	Facilitators	**Availability of resources—meds, equipment, and diagnostics**	Captures facilitators related to the presence and accessibility of essential medications, equipment, consumables, and diagnostic services required for effective triage and patient management. This includes the provision of adequate supplies, functional equipment, and the establishment of dedicated pharmacies, laboratories, or diagnostic areas that support timely assessment and treatment. Facilitators describing reliable access to these resources, to ensure continuity and quality of care during triage, fall under this theme.
		**Sustainable financing**	Captures facilitators related to the availability and stability of financial resources required to establish, operate, and sustain triage systems. This includes the presence of dedicated funding plans, institutional or governmental budget allocation, and financial mechanisms that ensure continuity beyond initial implementation. Facilitators describing secure and predictable funding that supports long-term maintenance, staffing, training, and resource replenishment fall under this theme. Facilitators focusing on the leadership support and commitment to provide such funds fall under "Leadership commitment".
		**Safe environment**	Captures facilitators related to the presence of measures, systems, and protocols that ensure the safety and security of staff within triage and emergency care settings. This includes the establishment of security procedures, presence of security personnel, and protocols for managing aggression or violence. Facilitators describing initiatives that promote a physically and psychologically safe working environment for staff fall under this theme.
		**Enabling work environment**	Captures facilitators related to supportive organizational and physical conditions that promote effective triage implementation and staff performance. This includes fair workload distribution, adequate remuneration, and recognition and motivation mechanisms. Facilitators describing positive workplace culture and systems that empower staff to perform efficiently and with confidence fall under this theme.
		**Surge planning**	Captures facilitators related to the presence and operationalization of facility-level plans, protocols, and systems to anticipate, prepare for, and respond to a surge in patient volume or acuity.
	Barriers	**Implementation cost**	Captures barriers related to the financial burden associated with establishing or maintaining triage systems. This includes costs linked to procuring equipment and materials, training staff, adapting infrastructure, or sustaining triage operations over time. Barriers related to the unavailability of funds to cover implementation costs are captured under "Unsustainable financing".
		**Lack of policy support**	Captures barriers related to the absence, or inadequacy of policies, regulations, or mandates that promote the implementation of standardized triage systems. This includes the lack of mandates to implement standardized triage and of national, regional, or facility-level policies and plans that define triage standards, responsibilities, or accountability mechanisms. This also includes the lack of unified national triage protocol. Barriers describing policy gaps or the absence of supportive governance structures necessary to institutionalize triage practices fall under this theme.
		**Poor Integration Within the Hospital**	Captures barriers related to weak coordination and linkages within hospital departments. This includes poor communication and collaboration between hospital departments and lack of communication during handover. Barriers describing fragmentation or inefficiencies in system components within the hospital that disrupt continuity of care and overload triage services fall under this theme.
		**Inefficient infrastructure and patient flow**	Captures barriers related to physical and structural limitations that hinder effective triage implementation and patient movement. This includes the absence of dedicated triage areas, poorly designed layouts, bidirectional or congested flow of patients, and the use of clinical spaces for non-clinical purposes such as storage. Barriers describing overcrowding, bottlenecks, long waiting times before triage, or inadequate spatial organization that impede timely assessment, privacy, and overall efficiency of triage processes fall under this theme. Barriers referring to lack of hygiene or patients family spaces and bathrooms facilities also fall under this theme.
		**Unsupportive work environment**	Captures barriers related to challenging or unsafe working conditions that negatively affect triage implementation. This includes excessive workload and patient volume, lack of support systems, and organizational cultures characterized by blame or fear rather than learning and collaboration. Barriers describing stressful, unstable, or unsafe environments that contribute to staff fatigue, reduced performance, and low morale fall under this theme.
		**Poor teamwork and task-sharing**	Captures barriers related to weak collaboration, communication, and role distribution among triage staff. This includes unclear responsibilities, limited delegation, and an overreliance on physicians for tasks that could be shared with or performed by nurses or other team members. Barriers describing the absence of effective teamwork, poor communication within the triage team, and bottlenecks resulting from inadequate task-sharing or empowerment fall under this theme. Barriers related to deficient patient identification such as the lack of patient identification bands also fall under this theme.
		**Unstable staffing structure**	Captures barriers related to staffing shortages, instability, and lack of continuity in personnel responsible for triage or emergency care. This includes high staff turnover, reliance on rotating rather than fixed staff assignments, and inadequate staffing levels and supervision that compromise consistency and accountability. Barriers describing frequent changes in personnel, insufficient human resources, unclear staff responsibilitirs, or the absence of structured staffing policies that ensure stable and experienced triage teams fall under this theme.
		**Inadequate resources and equipment**	Captures barriers related to the lack, insufficiency, or poor functionality of essential resources required for effective triage. This includes shortages or unavailability of medical equipment, consumables, including stationery, and medications, as well as limited access to diagnostic tools and testing capacity. Barriers describing broken, outdated, or inadequate supplies, and insufficient physical or material resources that hinder timely and accurate triage, fall under this theme.

		**Inadequate safety protocols**	Captures barriers related to the absence or weakness of safety and security measures designed to protect healthcare providers within triage and emergency care settings. This includes the lack of established protocols or procedures to manage aggression, violence, or other safety threats. Barriers describing insufficient security presence, inadequate staff training on de-escalation, or lack of institutional policies to ensure a safe working environment fall under this theme.
		**Inadequate surge planning**	Captures barriers related to the absence or weakness of policies, protocols, and preparedness measures for managing patient surges or mass casualty incidents (MCIs). This includes lack of structured plans, defined roles, and coordination mechanisms to guide triage and resource allocation during high-demand or crisis situations. Barriers describing limited institutional readiness, absence of surge or disaster response protocols, and insufficient integration of triage into emergency preparedness frameworks fall under this theme.
		**Unsustainable financing**	Captures barriers related to the lack of stable or long-term financial support necessary for establishing, operating, and maintaining triage systems. This includes absence of dedicated funding streams, reliance on short-term or project-based resources, and inadequate budget allocation for ongoing needs such as staffing, training, and equipment. Barriers describing financial instability that threatens the continuity, scalability, or sustainability of triage implementation fall under this theme.
		**Tecnnology limitations**	Captures barriers related to the absence, inadequacy, or poor functionality of technological systems that support triage implementation. This includes unreliable digital infrastructure, limited access to electronic tools or devices, and lack of technical support. Barriers describing challenges with e-triage platforms, issues with using apps or electronic devices such as iPads in specific triage systems, or communication technologies including poor network that hinder efficiency and accuracy in triage processes fall under this theme. Barriers related to data entry systems are captured under "inadequate documentation".
**Characteristics of Individuals**	**Facilitators**	**Stakeholder engagement and partnership**	Captures facilitators related to the active involvement and collaboration of key actors and organizations in the implementation of triage. This includes engagement of stakeholders as well as partnerships between institutions or organizations that provide technical support, resources, or coordination. Facilitators describing collaboration or support from external partners, including NGOs, international organizations, or donor agencies, are also captured under this theme.
		**Positive staff perception and attitude**	Captures facilitators related to staff's favorable perceptions, motivation, and openness toward triage implementation and emergency care improvement. This includes belief in the usefulness and value of triage, enthusiasm for adopting new systems, and a proactive desire to contribute to change. Facilitators describing motivated, engaged, and receptive staff who view triage as beneficial to patient care and workflow fall under this theme.
		**Leadership commitment**	Captures facilitators related to the active support, engagement, and endorsement of triage implementation by institutional and departmental leaders. This includes visible leadership buy-in, allocation of resources, and encouragement of staff participation, as well as direct involvement in planning, oversight, and problem-solving. Facilitators describing leadership support for specific implementation components—such as training, monitoring, or resource mobilization—also fall under this theme.
		**Supporting staffing and supervison**	Captures facilitators related to staffing structures and arrangements that enable effective triage implementation. This includes policies that promote stable staffing—such as assigning fixed rather than rotating staff—as well as flexible task-sharing and clear role definitions that improve workload distribution and accountability. Facilitators describing the presence of experienced or senior supervision in triage areas also fall under this theme.
	**Barriers**	**Community mistrust and poor communication**	Captures barriers related to the lack of community understanding, acceptance, or trust in triage processes. This includes misconceptions about how prioritization decisions are made, leading to perceptions of unfairness, partiality, or discrimination, as well as poor communication between healthcare providers and community members. Barriers describing limited public awareness of triage principles, misinterpretation of prioritization criteria, or the belief that one's own patient is more deserving of care fall under this theme.
		**Low staff motivation and engagement**	Captures barriers related to decreased enthusiasm, commitment, or willingness among staff to engage with triage implementation. This includes resistance to change, or viewing triage as an additional burden or as conflicting with more important tasks as actual patient care. Barriers describing general demotivation due to high workload, stressful or unsupportive work environments, low job satisfaction, or declining enthusiasm over time fall under this theme. Barriers related to bad work environment or circumstances that may lead to low staff motivation are captured under "Unsupportive work environment".
		**Low staff confidence and buy-in**	Captures barriers related to staff uncertainty about their ability to perform triage effectively and doubts regarding the value or effectiveness of the triage system itself. This includes low self-esteem or uncertainty that they are well-trained and qualified to make decisions when implementing triage.
		**Limited leadership commitment**	Captures barriers related to insufficient engagement, support, or prioritization of triage implementation by institutional or departmental leaders. This includes lack of visible leadership involvement, inadequate resource allocation, and absence of guidance or oversight during planning and execution. Barriers describing weak managerial support, limited advocacy for triage, or lack of leadership support for key components such as staff training and capacity building also fall under this theme.
		**Limited stakeholders engagement**	Captures barriers related to the insufficient involvement of key stakeholders in the planning, implementation, or evaluation of triage systems. This includes lack of consultation, coordination, or communication with stakeholders. Barriers describing limited collaboration, minimal ownership, or weak participation of relevant actors that hinder buy-in, alignment, and sustainability of triage implementation fall under this theme. Lack of community engagement leading to lack of awareness and community acceptance of triage is

Implementation Process	Facilitators	**Clear protocols and processes**	Captures facilitators associated with having well-defined and consistently applied protocols and procedures. This theme includes the presence of visible guidelines, flowcharts, or signage that clarify each step of the triage process, ensure standardization of patient assessment and prioritization, and support effective communication and coordination among staff. Processes to keep the triage area clean, organized, and stocked are also captured within this theme.
Implementation Process	Facilitators	**Structured implementation planning and readiness**	Captures facilitators related to thorough preparation and organized planning preceding or accompanying triage implementation. This includes conducting baseline assessments, feasibility studies, and pilot testing to understand the local system and tailor the system if needed; developing a structured implementation plan guided by a framework; and adopting phased rollout strategies that allow for adaptation and learning. Facilitators describing staff orientation to the triage process and objectives, or the presence of an existing triage or organizational system that provided a foundation for introducing a new one, also fall under this theme.
Implementation Process	Facilitators	**Capacity buidling and training**	Captures facilitators related to the availability and quality of training initiatives that strengthen staff competence and confidence in triage. This includes structured in-service and refresher trainings, Training of Trainers (ToTs), and the use of training manuals or other supportive materials. Facilitators describing training that accommodate staff schedules, involves interprofessional participation or non–healthcare workers where appropriate, and incorporates broader professional skills such as leadership, communication, and teamwork—all contributing to more effective triage implementation—fall under this theme. Facilitators referring to the use of e-trainings are included under this theme.
Implementation Process	Facilitators	**Robust implementation support structure**	Captures facilitators related to the elements of the implementation process. It includes organizational and human structures established to guide, coordinate, and sustain triage implementation. This theme focuses on the structural and functional aspects of implementation support—such as the presence of dedicated implementation teams, data collection teams, and advisory or technical working groups, and designating onsite mentors or champions to guide activities. Facilitators describing ongoing onsite support, mentorship, and structured oversight mechanisms that provide leadership, coordination, and accountability throughout the implementation process fall under this theme.
Implementation Process	Facilitators	**Standardized documentation and reporting**	Captures facilitators related to consistent and structured documentation practices that support effective triage implementation, monitoring, and sustainability. This includes the use of standardized triage forms, reporting templates, and record-keeping systems that promote accuracy, completeness, and continuity of information. Facilitators describing robust documentation processes that enable data tracking, performance evaluation, and quality improvement within triage systems fall under this theme.
Implementation Process	Facilitators	**Continuous M&E and Quality Improvement**	Captures facilitators related to ongoing monitoring, evaluation, and continuous quality improvement in triage. This includes the use of regular feedback loops, performance reviews, and structured mechanisms to assess implementation progress and identify areas for refinement. Facilitators describing continuous assessment and adaptation processes that strengthen accountability, learning, and overall triage effectiveness fall under this theme.
Implementation Process	Facilitators	**Digital and e-triage systems**	Captures facilitators related to the use of electronic, digital, or mobile technologies to support triage processes. This includes the implementation of e-triage platforms, mobile applications, and other digital tools—such as the use of smartphones or tablets—that enhance data entry, communication, and decision support. Facilitators describing digital innovations that improve triage accuracy, timeliness, documentation, or coordination across care levels fall under this theme.
Implementation Process	Barriers	**Inefficient hospital processes**	Captures barriers related to inefficient or defective operational procedures that hinder the smooth implementation of triage. This includes flawed hospital admission or payment processes that create access blocks, delays, or administrative bottlenecks. Barriers describing systemic process weaknesses and inefficient workflows that disrupt patient flow, impede timely care, or reduce the effectiveness of triage implementation fall under this theme.
Implementation Process	Barriers	**Inadequate documentation**	Captures barriers related to deficient or inconsistent documentation practices that hinder effective triage implementation, monitoring, and improvement. This includes incomplete or inaccurate record-keeping, lack of standardized triage forms or reporting systems, and absence of reliable data to support performance tracking and evaluation. Barriers describing weak documentation processes that limit accountability, quality assurance, and the ability to identify areas for system improvement fall under this theme.
Implementation Process	Barriers	**Inadequate implementation planning**	Captures barriers related to inadequate preparation and planning for triage implementation. This includes the absence of situational analysis, baseline assessment, or pilot testing prior to rollout, as well as lack of a structured or evidence-informed implementation plan. Barriers describing limited planning, insufficient assessment of feasibility, or failure to anticipate contextual challenges that affect the success and sustainability of triage systems fall under this theme.
Implementation Process	Barriers	**Insufficient training and mentorship**	Captures barriers related to the absence or inadequacy of training and ongoing mentorship necessary for effective triage implementation. This includes limited opportunities for staff to develop triage knowledge and skills, lack of refresher or in-service training, and absence of structured mentorship or onsite support. Barriers describing insufficient guidance, inadequate skill-building, or lack of onsite mentorship that leave staff unprepared or uncertain in performing triage fall under this theme. Barriers to training conduction like limited resources, time for training, and digital illetracy are included under this theme.

		**Weak monitoring and evaluation**	Captures barriers related to the absence or inadequacy of systems and plans for monitoring and evaluating triage implementation and performance. This includes lack of defined indicators or structured feedback processes to assess progress and inform improvement. Barriers describing limited oversight, absence of routine performance reviews, or weak integration of monitoring and evaluation into implementation frameworks fall under this theme. *Lack of accountability is captures under this theme.* Barriers related to lack of data collection mechanisms are captured under "Inadequate documentation".

This list has been refine to included iteratively the barriers and facilitators reported in the included studies. It is the the one that was used for analysis.
