# Supplementary material for: Identifying barriers and facilitators to implementing triage systems in low- and middle-income countries: A scoping review
Source: PLOS Glob Public Health. 2026 Sep 29;6(9):e0007378. doi: 10.1371/journal.pgph.0007378 (PMC13623090; doi:10.1371/journal.pgph.0007378)
Supplement: Supplementary file 3 [file pgph.0007378.s005.pdf]

S4 Table. Domain–Factor Definitions and Examples

Domain	Definition*	Factor	Definition as a facilitator	Definition as a barrier	Examples
Innovation (Triage Tool and Associated Process)	The degree to which triage protocols are evidence-based, adaptable to local contexts, easy to use and feasible to implement in resource-constrained settings.	Availability of a standardized triage tool	An acuity-based triage tool is available and formally adopted by the facility.	Lack of a standardized tool whereby providers use multiple tools inconsistently.	A facility uses a single triage scale for all patients, versus a facility where providers rely on individual clinical judgment.
		Evidence-based and acuity-based design	Triage tool is grounded in evidence and clearly links patient assessment to urgency.	Triage tool is arbitrary or not clinically meaningful.	The use of a triage scale aligned with international guidance versus questioning the validity of locally created tools.
		Clarity of categories and decision rules	Triage categories and criteria are clearly defined and easy to distinguish.	Categories overlap or are difficult to interpret.	Providers easily differentiate between urgent and non-urgent cases versus frequent disagreement about category assignment.
		Simplicity and ease of use	The triage tool is easy to use in real-time patient assessment allowing fast, consistent, and safe triage results.	Triage tool requires specialized training or equipment (beyond triage), requires many steps, or involves ambiguous criteria that make triage more difficult to apply safely.	A color-coded triage system with clear and easily defined thresholds that can be applied quickly at the point of care versus one that a multi-step scoring system that requires calculations or detailed interpretation by a specialist to complete.
		Trainability across cadres of providers	Triage tool can be taught to competence through brief, structured training that allows for a wide range of providers to perform triage.	Triage tool requires complex skills training, high baseline clinical expertise, or advanced judgment, limiting triage competence to senior staff.	A triage process that can be taught to and carried out by a team member with minimal training (e.g. nursing or medical student) vs. a proces that requires the judgement of a trained specialist (eg. Emergency Physician or Cardiologist interpretation of an electrocardiogram)
		Adaptability to local context	Triage tool can be adapted to patient volume, staffing, and resources without losing core structure and function.	Triage tool design is rigid and thereby limits adaptability to the local context.	Triage tool that incorporates vital signs that can be gathered with or without specialized equipment (e.g. Heart Rate, Resp Rate) vs one that requires specialized equipment (e.g. Oxygen Saturation)
		Integration with downstream processes	Triage tool guides to clear next steps after triage.	Triage decisions unrelated to other clinical processes and do not translate into patient-related actions.	Triage level designation is linked to clear actions (e.g. RED or level 1 / high acuity) patients taken to designated resuscitation area equipped with specialized equipment (monitors) or additional staffing to care for higher acuity case
		Cost of implementation and maintenance	Costs are low or manageable relevant to the implementing facility.	Training, materials, needed space or equipment, or supervision costs are prohibitive.	Use of open-source tools and training (e.g. WHO/ICRC/MSF Inter-Ageny Triage Tool) vs specialized or proprietary triage systems whose cost may make it available only to highly reesourced faciliites
		Availability of training and reference materials	Standardized, accessible training materials exist.	Materials are absent, outdated, or difficult to access or translate.	Use of job aids, posters, or digital modules which are consistent versus inconsistent or informal training.
		Documentation requirements	Documentation is simple and aligned with routine records.	Documentation is burdensome or duplicative.	Ability to quickly record triage level and for that triage designation to be reflected in the clinical documentation or health information system to flag acuity level and guide resource allocation
Implementation Process	How triage is introduced through structured planning and implementation, champions are engaged, staff are trained, documentation is maintained, and feedback loops are established to adapt and sustain practice.	Pre-implementation assessment	Planning integrates baseline assessment to consider local context in the plan.	The implementation plan does not consider the pre-existing conditions.	Assessing existing staffing, training, and space before rollout versus one-size-fits-all implementation
		Structured planning	Roles, timelines, and steps are defined, often informed by an implementation framework.	Implementation is ad hoc with no defined plan, identified steps, and roles.	Clear rollout plan versus informal, inconsistent adoption of triage
		Team composition and engagement	All relevant groups are involved in the planning and implementation.	Key actors are excluded from planning and implementation.	Inclusion of clinicians, security, and administrative staff ensures that everyone understands acuity based triage and able to message consistently with patients and families versus training that only focuses on clinicians and fails to garner support/buy-in from other team members needed to enforce triage system
		Training and refresher strategies	Ongoing training is part if continuous medical education scheme.	There is lack of refresher training in the context of frequent staff turnover.	Triage training occurs at time of onboarding staff and then repeated/available at regular intervals and in many formats (on shift, self-paced, formal classroom) vs system that only occurs at time of hiring or certain time of year. In the latter case, high staff turnover leads to loss of group knowledge regarding triage.
		Champions and local leadership	Local champions are identified and drive triage uptake.	No one is designated and empowered to implement the process.	One team member designated to coordinate trainings as core responsibility rather than as added mandate to full responsibilities or diffusely held responsibility by "everyone" that makes accountability difficult
		On-site support and supervision	Implementers receive follow-up support during implementation phases.	No support is available and arising issues go unaddressed.	Mentorship visits and supervision of early implementation phase versus unresolved provider challenges and concerns.
		Monitoring and evaluation	Indicators are identified and tracked with a plan in advance.	Performance is not reviewed or when issues are identified are not tracked.	Clinical audit system in place to randomly sample records for assessment of triage level to identify under/over triage and provide feedback to team members
		Use of quality improvement	Implementation is iterative.	Adaptation and continuous improvement is discouraged.	PDSA cycles used to refine triage versus rigid adherence despite issues.
Inner Setting (Facility and Organizational Context)	The role of person/community expectations, healthcare system funding, national health policies and laws, international guidelines, integrated linkages with prehospital and primary care systems, and the impact of large-scale or unanticipated events that may disrupt implementation or delivery of the innovation in supporting or hindering triage uptake.	Formal institutional endorsement	Triage is supported by hospital policies or directives.	Triage is optional or inconsistently enforced.	A written departmental or facility policy requiring triage supported by departmental / hospital leadership.
		Emergency department layout and infrastructure	Physical space supports triage and patient flow including clearly allocated spaces for triage, management of different acuity levels, and waiting rooms.	Space constraints limit triage visibility or safety.	Clear designation of clinical area with assignment of space, physical and human resources that correspond to needs of triage acuity level
		Availability of supporting clinical protocols	Standardized protocols exist to guide care after triage.	Triage decisions are not supported by standardized treatment protocols and pathways.	Clear standardized protocols for managing high-acuity patients versus delays or inconsistency of management after triage. (e.g. RED/High acuity area with increased space staffing and equipment for critically ill patients or GREEN low-acuity area that allows for multifunctional space (e.g. treatment chairs/rooms) that allow for the care of many different types of patients in same area (e.g. basic exam, laceration repair, medication administration)
		Security and safety measures	Security measures are in place to allow staff to feel protected while enforcing triage decisions.	Security measures are absent, thereby discouraging staff from enforcing triage due to safety concerns.	Presence of trained security staff and security policies to support triage and help govern access and patient flow.
		Financial support and sustainability	Sustainable funding is available to support training, supervision, and triage materials.	Triage relies on short-term or external funding only.	Budgeted refresher training versus loss of capacity when initial implementation is complete or when staff rotate to other parts of the hospital
		Staffing policies and task sharing	Staffing models, shift patterns, and task-sharing arrangements ensure continuous triage coverage using available staff.	Staffing practices, rotation, or lack of task-sharing result in gaps or inconsistency in triage delivery.	Staffing models that assign dedicated triage coverage using available cadres versus reliance on rotating providers without protected triage roles.
		Scope of practice and institutional endorsement of triage roles	Institutional rules or norms clearly define which provider cadres are permitted to perform triage.	Scope-of-practice expectations are unclear or restrictive, limiting who is permitted to perform triage regardless of service needs.	Facilities where nurses or clinical officers are formally recognized as permitted to perform triage versus settings where triage is informally restricted to physicians despite service needs.
		Staffing stability and turnover	Staffing is relatively stable with supporting retention strategies.	High turnover undermines training and continuity.	Maintenance of a core fixed cadre in the emergency department and frequent refresher training vs emergency units who rely on majority rotational staff where triage training and experience lost when staff rotates off to other services.
		High workload and patient flow	Patient volume and flow are manageable, allowing providers to perform triage and re-triage consistently.	High patient volume, overcrowding, or poor flow without ability to pull in additional resources	Barrier could be when high patient flow ovewhelms local resources resulting in a "local disaster" if there is no plan in place to pull in additional resources or transition to different triage system (e.g. mass incident triage) temporarily until flow abates.
		Stressful work environment	The work environment supports provider well-being and allows work to be performed under manageable stress levels.	High stress, frequent conflict, lack of appreciation or motivation, or emotional strain discourage providers from engaging in triage or maintaining triage quality.	A supportive environment may involve triage being conducted in an area with several providers nearby to support, with administrative staff available to handle non-clinical questions, and/or with security available to deal with potential escalation or violence instead of an environment where a single provider is left alone "out front" to deal with large numbers of patients and families.
		Internal communication and coordination	Departments communicate and coordinate patient care based on triage decisions.	Triage information is not shared or respected.	Smooth handover between triage and treatment teams with clear guidance based on triage level
Outer Setting (Health System and External Environment)	Organizational mission and culture, internal communication, resource availability and implementation costs, hospital policies and processes, the security of the environment, and the layout and infrastructure of the hospital and department.	National policies and guidelines	National guidance supports standardized triage.	National guidance is absent or conflicting.	National emergency care guidelines endorsing triage with compliance measured by governing bodies
		Regulatory and legal environment	Regulations protect providers implementing triage.	Legal uncertainty discourages enforcing triage decisions.	Clear triage-related regulations versus fear of blame for triage decisions.
		External stakeholder support	Professional bodies or partners endorse triage.	External priorities conflict with implementation.	Clear support from external stakeholders including departmental, facility, and ministry leadership help to facilitate triage through professional support, resource allocation and communication to patients, families and staff
		Community and patients engagement	Public messaging orient communities about triage and its goals and benefits.	Limited awareness about triage lead to resistance at the point of care.	Public awareness campaigns or routine exposure to triage in healthcare settings can support triage implementation. Examples include public awareness campaigns in print and electronic media, posters in triage and waiting areas, short videos to play on monitors in patient facing areas to familiarize patients and families with triage rationale and process
		Health system financing mechanisms	Financing to support emergency care systems and functions is available and sustainable.	There is limited or unsustainable financing to support emergency care systems and functions.	Triage training is integrated into routine / core activities and financed accordingly vs triage training that is reliant on donor funding that is not sustained
		Integration across the care continuum	Triage aligns with prehospital, referral, and primary care systems.	Systems are fragmented and operate in silos.	Triage principles can be integrated across the care continuum including prehospital services (prehospital notification re critical patients) or assessment of facility capacity by administrative staff (incorporation of number of patients by acuity into assessments needed for hospital staffing, for example)
Individuals (People involved in or affected by triage)	Providers competence level and attitudes toward triage, confidence in using tools, perceptions of their role within triage, the contributions of all members of the implementation teams and the leadership support in enabling effective implementation.	Provider knowledge and skills	Providers are trained and confident to apply triage and make triage-related decisions.	There are persistent training gaps or providers are not confident in their abilities to appropriately triage	Triage process will break down if providers are forced into triage without proper training or have impression that their triage assessments are only assessed in one direction (e.g. "better be safe than sorry" approach resulting in overtriage and overwhelming resources)
		Providers' attitudes toward triage	Triage is valued as a practice that has a positive impact on patient outcomes and emergency care service.	Triage is perceived as unnecessary, unfair, or less valuable than individual providers' decision based on their own experience.	Need for constant appraisal of team attitudes towards triage and identification of self-perceived barriers will help ensure that staff have buy-in and see triage as useful to providing quality emergency care
		Role understanding and accountability	Individuals involved in emergency care clearly understand their role in the triage process and perceive responsibility for carrying out triage-related tasks.	Individuals are uncertain about whether triage is part of their role, leading to hesitation, duplication, or omission of triage activities.	Providers who understand when and how they are expected to perform triage or re-triage versus situations where multiple staff assume someone else is responsible for triage.
		Provider motivation, satisfaction, and sense of accomplishment	Providers feel motivated, satisfied, or accomplished from their jobs in general.	Providers are demotivated, disengaged and dissatisfied.	Providers expressing pride in providing emergency care and positively impacting patient outcomes versus providers reporting job frustration or disengagement.
		Leadership support	Leaders visibly support triage implementation and endorsement.	Leadership is not engaged in triage implementation or does not endorse triage implementation.	ED leadership reinforcing triage practices versus lack of support or follow-up on triage implementation.
		Non-clinical staff awareness and involvement	Security, admin, and support staff are aware of triage concepts and are involved.	Security, admin, and support staff are excluded and are not engaged with triage practices.	Security de-escalating conflicts, thereby aiding in patient flow versus escalating conflicts causing disruption of clinical duties.
		Patient and family perceptions	Patients and families perceive triage decisions as fair and appropriate during their care encounter.	Patients or families challenge prioritization or express distrust during triage interactions.	Families accepting longer wait times for low-acuity cases versus demanding immediate attention despite lower urgency.

* These definitions were adpated from the CFIR constructs definitions to be specifically applicable to triage implementation.
This list has been developed based on the reported barriers and facilitators and further refined to be as comprehensive as possible and include all potential CFIR-mapped triage factors. It includes definitions of the factors illustrated in the figure and explain them in terms of potential barriers or facilitators or both.
