# Supplementary material for: Identifying barriers and facilitators to implementing triage systems in low- and middle-income countries: A scoping review
Source: PLOS Glob Public Health. 2026 Sep 29;6(9):e0007378. doi: 10.1371/journal.pgph.0007378 (PMC13623090; doi:10.1371/journal.pgph.0007378)
Supplement: Supplementary file 4 [file pgph.0007378.s003.docx]

**S2 Table. Included Studies**

| Title | First Author and Year of Publication | Country/ies | | | Study Design | | Triage Tool | Population | Intervention/ Exposure | Sample Size | Reported Domains | Reported Barriers and Facilitators | Quality Appraisal (Risk of Bias) |
| --- | --- | --- | --- | --- | --- | --- | --- | --- | --- | --- | --- | --- | --- |
| A complex intervention to improve implementation of World Health Organization guidelines for diagnosis of severe illness in low-income settings: a quasi-experimental study from Uganda | Cummings et al, 2017 | Uganda | | | Quantitative | | WHO Quick Check | Adult and adolescent patients (age ≥ 14 years) admitted to general medical wards through the casualty department at four inpatient health facilities (three general hospitals and one community health center) in western Uganda. | Intervention: The Severe Illness Management System (SIMS) platform, a multi-modal quality improvement program. It included clinical mentoring, collaborative improvement meetings, clinical performance audit and feedback, and clinical mentoring, following formal training on WHO "Quick Check" guidelines. | 5,759 patients (1,633 in the pre-intervention period and 4,126 during the intervention period). | - Implementation Process - Inner Setting - Individuals | Barriers   - Inefficient infrastructure and patient flow - Inadequate resources and equipment - Insufficient training and mentorship   Facilitators   - Structured implementation planning and readiness - Integrated teamwork practice - Stakeholder engagement and partnership - Capacity building and training - Efficient layout and infrastructure - Robust implementation support structure - Leadership commitment - Supporting staffing and supervision - Standardized documentation and reporting - Continuous M&E and Quality Improvement - Sustainable financing | Medium Risk |
| A continued professional development nursing partnership in a remote Bolivian hospital | Rivasplata et al, 2021 | | | Bolivia | | Quantitative | Manchester Triage System (MTS) 5-points triage system | Primary health professionals (including nurses, physicians, and auxiliary nurses) working in the emergency department of a district hospital in the Bolivian Amazon Basin. | Intervention: An evidence-based triage training program based on the Manchester Triage System (MTS), which included didactic sessions, role-play, simulation, and a subsequent 4-week triage pilot. | 19 health professionals. | - Implementation Process - Inner Setting - Innovation - Individuals | Barriers   - Complex, deficient and unclear triage tools and protocols - Inadequate resources and equipment   Facilitators   - Clear protocols and processes - Structured implementation planning and readiness - Contextual and cultural adaptability - Integrated teamwork practice - Stakeholder engagement and partnership - Capacity building and training - Robust implementation support structure - Leadership commitment - Continuous M&E and Quality Improvement | Medium Risk |
| A mixed-methods study on evaluating an updated, francophone version of ETAT+ training in Madagascar | Galatsch et al, 2022 | Madagascar | | | Mixed Methods | | Adapted Malagasy version of ETAT+ | Malagasy health professionals, specifically paediatricians, physicians, nurses, and midwives, working in paediatric and emergency departments across six health facilities in four regions of Madagascar. | Intervention: An updated Malagasy version of the Emergency Triage Assessment and Treatment Plus (ETAT+) pilot training course. | 12 participants completed the training and quantitative evaluation, and 5 of these participants were randomly selected for follow-up qualitative interviews. | - Implementation Process - Inner Setting - Innovation - Individuals | Barriers   - Poor contextualization - Inadequate resources and equipment - Limited leadership commitment - Insufficient training and mentorship - Unsustainable financing   Facilitators   - Integrated teamwork practice - Stakeholder engagement and partnership - Positive staff perception and attitude - Capacity building and training - Robust implementation support structure - Leadership commitment - Digital and e-triage systems - Enabling work environment | Low Risk |
| A quality improvement study of the emergency centre triage in a tertiary teaching hospital in northern Ethiopia. | Abdelwahab et al, 2017 | | | Ethiopia | | Quantitative | SATS | Adult emergency patients (over the age of 18 years) and ED triage nurses and physicians at the emergency centre (EC) of Ayder Comprehensive Specialized Hospital (ACSH), | Exposure: An adapted version of the South African Triage Scale (SATS), employing a Triage Early Warning Score (TEWS) and additional clinical discriminators, was already in place at the study site (introduced in 2012). This study evaluates nurse-led triage using the existing tool; no new intervention was delivered. | 251 patients were randomly selected for the study (with 107 charts retrieved and 45 triage forms included in the final analysis) | - Implementation Process - Inner Setting - Individuals | Barriers   - Low staff motivation and engagement - Inefficient infrastructure and patient flow - Unsupportive work environment - Poor teamwork and task-sharing - Poor hospital processes - Unstable staffing structure - Inadequate resources and equipment - Insufficient training and mentorship   Facilitators   - Capacity building and training - Efficient layout and infrastructure - Leadership commitment - Supporting staffing and supervision | Medium Risk |
| Adapting a novel emergency triage tool to a resource-limited hospital in Nepal | Weiner et al, 2025 | Nepal | | | Qualitative | | IITT | Clinical staff (doctors, nurses, and paramedics) working at the Dhulikhel Hospital Emergency Department (DHED) in central Nepal, and ED patients | Exposure: A qualitative co-design process explored staff perceptions of the existing (non-standardized) triage process and collaboratively drafted a proposed workflow to adapt the IITT. | 34 participants, representing over 80% of the clinical staff at the facility | - Implementation Process - Inner Setting - Innovation - Individuals | Barriers   - Complex, deficient and unclear triage tools and protocols - Poor Integration Within the Hospital - Inefficient infrastructure and patient flow - Poor teamwork and task-sharing - Inadequate resources and equipment - Insufficient training and mentorship - Inadequate safety protocols   Facilitators   - Simple and user-friendly tools - Clear protocols and processes - Contextual and cultural adaptability - Integrated teamwork practice - Positive staff perception and attitude - Capacity building and training - Supporting staffing and supervision - Availability of resources—meds, equipment, and diagnostics - Standardized documentation and reporting - Continuous M&E and Quality Improvement - Visual triage aids | Medium Risk |
| Application of queuing theory to optimize the triage process in a tertiary emergency care ("ER") Department | Moreno-Carrillo et al, 2019 | | | Colombia | | Quantitative | ESI | Adult patients requesting emergency care at the Hospital Universitario San Ignacio, a tertiary academic urban hospital in Bogotá, Colombia. The study specifically excluded gynecology and pediatric triages. | Intervention: The application of queuing (or “waiting line”) theory to determine the necessary number and distribution of nursing personnel in the triage area, followed by the implementation of staff schedule changes to reduce "pretriage" waiting times. | The study analyzed a total of 184,395 patient visits across two seven-month periods: 89,898 visits in the first (baseline) period and 94,497 visits in the second (post-intervention) period. | Implementation Process  Inner Setting  Individuals | Facilitators   - Integrated teamwork practice - Robust implementation support structure - Leadership commitment - Supporting staffing and supervision | Low Risk |
| Are we ready? Emergency unit capacity at selected district level hospitals in Lusaka Province, Zambia: barriers and facilitators for improving trauma care: a mixed methods approach | Machona et al, 2025 | Zambia | | | Mixed Methods | | N/A | Emergency unit healthcare providers (doctors, nurses, clinical officers) and hospital administrators at district-level health facilities in Lusaka Province, Zambia.  Patients presenting to district-level emergency units | Exposure: No triage intervention was delivered. The study used the WHO Hospital Emergency Assessment Tool (HEAT) alongside qualitative interviews to assess existing emergency trauma care capacity and identify barriers/facilitators. | 10 facilities (33 staff surveyed for HEAT; 35 staff interviewed). | - Implementation Process | Barriers   - Insufficient training and mentorship   Facilitators   - Clear protocols and processes - Efficient layout and infrastructure | Low Risk |
| Assessment of attitudes toward the emergency triage system in Belize | Genisca et al, 2020 | | | Belize | | Qualitative | ESI and ETAT | Pediatric Health care providers (HCPs), specifically physicians and practical and registered nurses, from the pediatric and accident and emergency wards at Karl Heusner Memorial Hospital Authority (KHMHA) and government health centers (GHCs) in Belize | Intervention: ETAT training was delivered directly by the study team to HCPs at KHMHA in February 2016. Focus groups were conducted immediately after and 1 year post-training to assess attitudes toward the training and the existing, heterogeneous triage systems. | 6 focus groups in total: 16 HCPs (7 physicians and 9 nurses) participated in the first set of focus groups (February 2016) and 25 HCPs (16 physicians and 9 nurses) participated in the second set (February 2017) | - Implementation Process - Inner Setting - Innovation - Individuals | Barriers:   - Complex, deficient and unclear triage tools and protocols - Poor contextualization - Inefficient infrastructure and patient flow - Poor hospital processes - Inadequate resources and equipment - Insufficient training and mentorship   Facilitators   - Structured implementation planning and readiness - Contextual and cultural adaptability - Integrated teamwork practice - Positive staff perception and attitude - Capacity building and training | Low Risk |
| Assessment of hospital-based adult triage at emergency receiving areas in hospitals in Northern Uganda. | Opiro et al, 2017 | Uganda | | | Quantitative | | Varied or non-existent depending on hospital | Health professionals (doctors, clinical officers/physician assistants, registered nurses, enrolled nurses, and nursing assistants) involved in the daily initial management of emergency patients in hospitals within the Acholi sub-region of Northern Uganda. | Exposure: No standardized triage system was in place across surveyed hospitals; practices varied or triage was absent depending on site. | 33 participants from 6 hospitals. | - Implementation Process - Inner Setting - Outer Setting - Innovation - Individuals | Barriers   - Complex, deficient and unclear triage tools and protocols - Lack of policy support - Unstable staffing structure - Insufficient training and mentorship   Facilitators   - Clear protocols and processes - National legislations and regulatory framework - Capacity building and training - Efficient layout and infrastructure - Leadership commitment - Supporting staffing and supervision | Medium Risk |
| Assessment of interrater reliability of the Emergency Severity Index after implementation in emergency departments in Jamaica using a learning collaborative approach | French et al, 2021 | | | Jamaica | | Quantitative | ESI | Emergency nurses and doctors at 19 accident and emergency department sites across Jamaica. | Intervention: Implementation of the Emergency Severity Index (ESI) triage tool supported by a learning collaborative approach, which included initial training followed by nine months of monthly webinars and expert case reviews. | 166 triage cases were submitted for review; the initial phase involved training 101 healthcare providers (57 physicians and 44 nurses). | - Implementation Process - Inner Setting - Innovation - Individuals | Barriers   - Complex, deficient and unclear triage tools and protocols - Poor contextualization - Low staff motivation and engagement - Unstable staffing structure - Inadequate resources and equipment - Insufficient training and mentorship - Tecnology limitations   Facilitators   - Capacity building and training - Continuous M&E and Quality Improvement | Low Risk |
| Assessment of knowledge and skills of triage amongst nurses working in the emergency centres in Dar es Salaam, Tanzania | Aloyce et al, 2013 | Tanzania | | | Quantitative | | N/A | Enrolled and registered nurses working in the emergency centres of the national hospital and three municipal district hospitals in Dar es Salaam, Tanzania  Acute patients presenting for emergency care at public hospitals | Exposure: No standardized triage system was described. The study assessed nurses' baseline knowledge and skills relevant to triage. | 4 facilities (60 nurses surveyed; 19 nurses observed). | - Implementation Process - Inner Setting - Innovation | Barriers   - Complex, deficient and unclear triage tools and protocols - Inadequate resources and equipment - Inadequate documentation - Insufficient training and mentorship | Medium Risk |
| Barriers and facilitators to providing facility-based emergency care in Addis Ababa, Ethiopia: A mixed methods study | Ambushe et al, 2025 | | | Ethiopia | | Mixed Methods | N/A | Eligible emergency care clinical providers and leaders, including experienced emergency nurses, permanently based emergency physicians, postgraduate emergency medicine registrars, emergency unit general practitioners (GPs), and administrators | Exposure: No specific triage tool was identified. The study examined barriers and facilitators to facility-based emergency care broadly. | 5 facilities (38 staff surveyed; 15 staff interviewed) | - Implementation Process - Individuals | Barriers   - Insufficient training and mentorship   Facilitators   - Supporting staffing and supervision | Low Risk |
| Capacity building for health care workers and support staff in Pediatric Emergency Triage Assessment and Treatment (ETAT) at primary health care level in resource limited settings: experiences from Malawi | Majamanda et al, 2021 | Malawi | | | Quantitative | | ETAT | Health care workers and support staff working at primary health care (PHC) facilities in the Blantyre and Chikhwawa districts of Malawi. | Intervention: A multi-faceted capacity-building program consisting of: a pediatric Emergency Triage Assessment and Treatment (ETAT) training package (adapted for both clinical and non-clinical staff); the provision of a minimum treatment package (emergency equipment, supplies, and drugs); and quarterly supportive supervisory visits. | 877 participants were trained (comprising 336 health care workers and 541 support staff). Additionally, the project monitored the triaging of 195,151 children during the implementation period. | - Implementation Process - Inner Setting - Outer Setting - Innovation - Individuals | Barriers   - Unstable staffing structure - Insufficient training and mentorship   Facilitators   - Contextual and cultural adaptability - Integrated teamwork practice - Stakeholder engagement and partnership - Effective referral and transport - Capacity building and training - Efficient layout and infrastructure - Robust implementation support structure - Leadership commitment - Supporting staffing and supervision - Availability of resources—meds, equipment, and diagnostics | Medium Risk |
| Developing emergency triage systems in Cambodia | Khan et al, 2020 | | | Cambodia | | Mixed Methods | A context-specific triage system was created using the input of a spectrum of local stakeholders. | Adult, pediatric, and maternity patients arriving for unscheduled visits, and the clinical staff (typically nurses) responsible for triage implementation at 35 public hospitals across nine Cambodian provinces. | Intervention: Implementation of a tailored, context-specific patient triage system, using elements from both ETAT and SATS, supported by a two-day training curriculum, quarterly quality improvement visits, and a standardized triage form. | 35 public hospitals; 3,250 patient chart audits conducted during 325 follow-up visits; and structured interviews with project staff. | - Implementation Process - Inner Setting - Innovation - Individuals | Barriers   - Inefficient infrastructure and patient flow - Unsupportive work environment - Unstable staffing structure - Inadequate resources and equipment - Limited leadership commitment - Limited stakeholders engagement - Weak monitoring and evaluation   Facilitators   - Structured implementation planning and readiness - Contextual and cultural adaptability - Stakeholder engagement and partnership - Capacity building and training - Robust implementation support structure - Leadership commitment - Availability of resources—meds, equipment, and diagnostics - Standardized documentation and reporting - Continuous M&E and Quality Improvement | Medium Risk |
| Effect of emergency nurses’ work stress, workload, and motivation on Emergency Severity Index implementation | Riu et al, 2024 | Iran | | | Quantitative | | N/A | Emergency department nurses employed for at least six months who have direct involvement in triage decision-making and are familiar with the Emergency Severity Index (ESI) system | Exposure: An ESI-based triage system was already implemented at the study site. This study examined the correlation between nurses' work stress, workload, and motivation and ESI use; no new intervention was delivered. | 72 ED nurses | - Inner Setting - Innovation - Individuals | Barriers   - Complex, deficient and unclear triage tools and protocols - Unsupportive work environment   Facilitators   - Positive staff perception and attitude | Medium Risk |
| Effective triage in the Pacific region: The development and implementation of the Solomon Islands Triage Scale | WANEFALEA et al, 2019 | | | Solomon Islands | | Quantitative | Solomon Islands Triage Scale (SITS) | Emergency department staff (doctors and nurses) and ED patient presentations at the National Referral Hospital in Honiara, Solomon Islands. | Intervention: Development and implementation of the Solomon Islands Triage Scale (SITS), a three-tier system based on the South African Triage Scale (SATS),. The intervention included staff training, revised patient flow pathways, and updated data management processes. | 10,905 patient presentations were included in the evaluation of the period (August–October 2017). 96 patient records were audited. | - Implementation Process - Inner Setting - Innovation - Individuals | Barriers   - Low staff motivation and engagement - Inefficient infrastructure and patient flow - Unsupportive work environment - Low staff confidence and buy-in - Inadequate resources and equipment - Limited leadership commitment   Facilitators   - Contextual and cultural adaptability - Integrated teamwork practice - Stakeholder engagement and partnership - Capacity building and training - Efficient layout and infrastructure - Robust implementation support structure - Leadership commitment - Supporting staffing and supervision - Safe environment | Low Risk |
| Emergency nurses' knowledge and experience with the triage process in Hunan Province, China | Hammad et al, 2017 | China (Changsha, Hunan Province) | | | Quantitative | | N/A | Emergency nurses working in 13 tertiary hospitals in Changsha, Hunan Province, China. | Exposure: No standardized triage tool was described. The study assessed nurses' knowledge of and experience with existing triage processes. | 193 emergency nurses completed the survey (from an initial sample of 300, representing a 64.3% response rate). | - Implementation Process - Individuals | Barriers   - Insufficient training and mentorship   Facilitators   - Clear protocols and processes - Capacity building and training - Robust implementation support structure   Leadership commitment | Low Risk |
| Emergency Triage Assessment and Treatment Plus (ETAT+): adapting training to strengthen quality improvement and task-sharing in emergency paediatric care in Sierra Leone. | Hands et al, 2021 | | | Sierra Leone | | Quantitative | ETAT+ | Clinical staff (primarily nurses, but also including doctors and community health officers) and paediatric patients at 14 regional and district government hospitals in Sierra Leone. | Intervention: An adapted Emergency Triage Assessment and Treatment Plus (ETAT+) programme. This included a five-day course delivered as a six-month programme of in-situ training and mentoring, combined with patient flow and service delivery improvements. | 727 nurses were trained (410 in 2017; 317 in 2018). The analysis included data from 20,711 paediatric admissions (6,651 in 2017; 14,060 in 2018) across participating hospitals. | - Implementation Process - Inner Setting - Outer Setting - Innovation - Individuals | Barriers   - Inefficient infrastructure and patient flow - Poor hospital processes - Unstable staffing structure - Inadequate resources and equipment   Facilitators   - Simple and user-friendly tools - Structured implementation planning and readiness - National legislations and regulatory framework - Stakeholder engagement and partnership - Capacity building and training - Efficient layout and infrastructure - Robust implementation support structure - Leadership commitment - Supporting staffing and supervision - Availability of resources—meds, equipment, and diagnostics - Standardized documentation and reporting - Continuous M&E and Quality Improvement | Low Risk |
| Evaluation of a digital triage platform in Uganda: A quality improvement initiative to reduce the time to antibiotic administration | Lee et al, 2020 | Uganda | | | Quantitative | | A digital triage platform that included a mobile application running on Nexus 6 Android phones (Motorola Mobility, Chicago, USA), combined with a pulse oximetry sensor attachment (LionsGate Technologies, Vancouver, Canada), to collect clinical symptoms | Children up to sixteen years of age presenting at the outpatient department of Holy Innocents Children’s Hospital in Mbarara, Uganda. | Intervention: A digital triage platform comprising a mobile application for patient prioritization (using clinical algorithms and vital sign data) and a computer-based dashboard for real-time patient tracking and resource allocation. | 11,220 children in total: 5,698 in the pre-implementation phase and 5,522 in the post-implementation phase. | - Implementation Process - Innovation - Individuals | Facilitators   - Contextual and cultural adaptability - Capacity building and training - Leadership commitment - Standardized documentation and reporting - Continuous M&E and Quality Improvement - Digital and e-triage systems | Low Risk |
| Explaining challenges of obstetric triage structure: A qualitative study | Fakari et al, 2020 | | | Iran | | Mixed Methods | N/A | Triage team members and key informants (including tri-  age performer, emergency specialist, gynaecologist, the charge of  the Department of Maternal Death, emergency department super-  visor, client and caregiver) at educational-therapeutic hospitals affiliated with Shahid Beheshti University of Medical Sciences in Tehran, Iran. | Exposure: No standardized obstetric triage tool was in place. The study explored structural challenges of existing obstetric triage practices. | 21 participants. | - Implementation Process - Inner Setting - Outer Setting - Innovation - Individuals | Barriers   - Complex, deficient and unclear triage tools and protocols - Poor contextualization - Poor System Integration / Fragmented System (Across Levels of Care) - Poor system integration/fragmented system - Community mistrust and poor communication - Low staff motivation and engagement - Inefficient infrastructure and patient flow - Unsupportive work environment - Unstable staffing structure - Inadequate resources and equipment - Inadequate documentation - Poor implementation planning - Insufficient training and mentorship - Limited stakeholders engagement   Facilitators   - Clear protocols and processes - Contextual and cultural adaptability - Integrated teamwork practice - Stakeholder engagement and partnership - Positive staff perception and attitude - Efficient layout and infrastructure   Community engagement and awareness | Low Risk |
| Exploring the effect of implementation and context on a stepped-wedge randomised controlled trial of a vital sign triage device in routine maternity care in low-resource settings | Vousden et al, 2019 | Ethiopia, Haiti, Sierra Leone, India, Zimbabwe, Zambia, Uganda, Malawi | | | Mixed Methods | | CRADLE Vital Signs Alert (VSA) | Healthcare providers (HCP) working in maternity care and pregnant or postpartum women (up to 42 days after delivery) across eight low- and middle-income countries: Ethiopia, India, Haiti, Malawi, Sierra Leone, Uganda, Zambia, and Zimbabwe. | Intervention: The CRADLE Vital Signs Alert (VSA) (a device measuring blood pressure and heart rate with a traffic light early warning system) and a corresponding educational training package delivered to "CRADLE Champions". | The process evaluation included 130 participants (via 36 interviews and 19 focus group discussions). 2,747 healthcare providers were trained across 286 facilities during the intervention period. | - Implementation Process - Inner Setting - Outer Setting - Innovation - Individuals | Barriers   - Poor System Integration / Fragmented System (Across Levels of Care) - Unstable staffing structure   Facilitators   - Simple and user-friendly tools - Clear protocols and processes - Structured implementation planning and readiness - Contextual and cultural adaptability - Integrated teamwork practice - Stakeholder engagement and partnership - Effective referral and transport - Capacity building and training - Robust implementation support structure - Leadership commitment - Supporting staffing and supervision - Availability of resources—meds, equipment, and diagnostics - Continuous M&E and Quality Improvement - Community engagement and awareness - Digital and e-triage systems - Safe environment - Visual triage aids - Enabling work environment | Low Risk |
| Factors influencing the implementation of obstetric triage: a qualitative study | Moudi et al, 2021 | | | Iran | | Qualitative | N/A | professionals (midwives, nurses, gynecologists, emergency medicine specialists, executive managers, and policymakers) and patients. | Exposure: No standardized obstetric triage protocol was in place. The study examined factors influencing implementation of obstetric triage. | 43 participants (37 professionals and 6 patients) | - Implementation Process - Inner Setting - Outer Setting - Innovation - Individuals | Barriers   - Complex, deficient and unclear triage tools and protocols - Poor contextualization - Poor Integration Within the Hospital - Poor System Integration / Fragmented System (Across Levels of Care) - Poor system integration/fragmented system - Community mistrust and poor communication - Low staff motivation and engagement - Inefficient infrastructure and patient flow - Unsupportive work environment - Low staff confidence and buy-in - Limited leadership commitment - Poor implementation planning - Limited stakeholders engagement   Facilitators   - Positive staff perception and attitude - Robust implementation support structure | Low Risk |
| Health worker perspectives of Smart Triage, a digital triaging platform for quality improvement at a referral hospital in Uganda: a qualitative analysis | Novakowski et al, 2022 | Uganda | | | Qualitative | | ETAT via Smart Triage | Health workers (including pharmacy dispenser, medical records officer, community linkage facilitators, nursing officers, clinical officers, and doctors) from the pediatric OPD, general in-patient ward, and nutritional ward at a regional referral hospital in Eastern Uganda. | Exposure: Smart Triage, a digital triaging platform, was already deployed at the study site. The study explored health workers' perspectives on the existing platform; no new intervention was delivered. | 15 health workers. | - Implementation Process - Inner Setting - Outer Setting - Innovation - Individuals | Barriers   - Unstable staffing structure - Low staff confidence and buy-in - Inadequate resources and equipment - Tecnology limitations   Facilitators   - Positive staff perception and attitude - Capacity building and training - Efficient layout and infrastructure - Robust implementation support structure - Standardized documentation and reporting - Community engagement and awareness - Digital and e-triage systems - Visual triage aids | Low Risk |
| Implementation of a novel three-tier triage tool in Papua New Guinea: a model for resource-limited emergency departments | Mitchell et al, 2020 | | | Papua New Guinea | | Editorial | IITT | Emergency Department (ED) staff, including emergency physicians, registrars, health extension officers (HEOs), nurses, and community health workers (CHWs). | Intervention: Implementation IITT, supported by a five-hour modular teaching program, clinical redesign, support material, the introduction of simple electronic registries, and hands-on mentorship. | 39 staff members participated in the evaluation survey (24 from Gerehu General Hospital (GGH) and 15 from Mount Hagen  Provincial Hospital (MHPH)). | - Implementation Process - Inner Setting | Barriers   - Poor Integration Within the Hospital - Inefficient infrastructure and patient flow - Unsupportive work environment - Poor teamwork and task-sharing - Poor hospital processes - Inadequate documentation | Low Risk |
| Implementation of a triage score system in an emergency room in Timergara, Pakistan | Dalwai et al, 2013 | Pakistan | | | Quantitative | | SATS | Triage staff and patients presenting to the emergency department of the Timergara District Headquarter (DHQ) Hospital in the Lower Dir district of Pakistan. | Intervention: Implementation of the South African Triage Scale (SATS). This process included staff training, infrastructure changes (creating a dedicated triage room), community awareness raising, and the use of the Triage Early Warning Score (TEWS) and discriminators. | 370 triage forms were evaluated (from an initial collection of 381 forms, with 11 excluded due to missing information). | - Implementation Process - Inner Setting - Outer Setting - Innovation - Individuals | Facilitators   - Contextual and cultural adaptability - Integrated teamwork practice - Stakeholder engagement and partnership - Efficient layout and infrastructure - Robust implementation support structure - Community engagement and awareness - Safe environment | MediumRisk |
| Implementation of the Interagency Integrated Triage Tool (IITT) in a rural emergency department: a qualitative study in Western Nepal | NAIR et al, 2025 | | | Nepal | | Mixed Methods | IITT | ED clinical staff including medical officers, health assistants, and certified medical assistants, and  pediatric and adult patients presenting for emergency and trauma care, at Bayalpata Hospital ED located in rural Nepal. | Intervention: Implementation of IITT including training, clinical tool tailoring, triage desk resignation and clinical redesign and flow, creating and displaying reference materials, and onsite mentorship. | 1 facility (20 clinical staff trained; 14 staff surveyed pre-implementation, 6 staff surveyed post-implementation, 22 patients/guardians surveyed post-implementation). | - Implementation Process - Inner Setting - Innovation | Barriers   - Complex, deficient and unclear triage tools and protocols - Inefficient infrastructure and patient flow - Unstable staffing structure - Inadequate resources and equipment   Facilitators   - Capacity building and training | Low Risk |
| Implementation of triage in the hospital emergency room of private hospital, Yogyakarta, Indonesia | Santosa et al, 2015 | Indonesia | | | Mixed Methods | | ATS | Patients and healthcare staff within the Emergency Room (ER) of PKU Muhammadiyah Hospital of Yogyakarta, Indonesia. The qualitative portion included "internal consumers" (medical directors, ER chiefs, doctors, nurses, and triage/information officers) and "external consumers" (patients and their families). | Exposure: The Australasian Triage Scale (ATS) was already in use at the study site; no new intervention was delivered. | 40 participants in total: a quantitative sample of 30 patients and a qualitative sample of 10 informants. | - Implementation Process - Inner Setting - Individuals | Barriers   - Lack of policy support - Inefficient infrastructure and patient flow - Inadequate resources and equipment - Inadequate documentation - Inadequate surge planning   Facilitators   - Structured implementation planning and readiness - Institutional and Hospital Policies - Capacity building and training - Leadership commitment - Availability of resources—meds, equipment, and diagnostics - Continuous M&E and Quality Improvement | Low Risk |
| Implementing a triage tool to improve appropriateness of care for children coming to the emergency department in a small hospital in India | Joshi et al, 2020 | | India | | | Quantitative | Locally adapted SATS | Paediatric patients and the healthcare staff (nurses, resident medical officers, and paediatricians) at the emergency department of Sitaram Bhartia Institute of Science and Research in Delhi, India. | Intervention: Implementation and adaptation of the SATS into a local paediatric triage tool. This included associated systemic changes such as staff training, increased nursing levels, feedback huddles, simplification of clinical records, and establishing local leadership, and training a restricted group of locum paediatricians. | N/A (Quality improvement initiative in 1 facility over 20 months) | - Implementation Process - Inner Setting - Innovation - Individuals | Barriers   - Low staff motivation and engagement - Unsupportive work environment - Poor teamwork and task-sharing - Poor hospital processes - Unstable staffing structure - Inadequate resources and equipment - Inadequate documentation - Insufficient training and mentorship - Tecnology limitations   Facilitators   - Simple and user-friendly tools - Clear protocols and processes - Structured implementation planning and readiness - Contextual and cultural adaptability - Integrated teamwork practice - Positive staff perception and attitude - Capacity building and training - Robust implementation support structure - Leadership commitment - Supporting staffing and supervision - Standardized documentation and reporting - Continuous M&E and Quality Improvement - Surge planning | Low Risk |
| Implementing the Emergency Severity Index triage system in Jamaican Accident and emergency departments | French et al, 2019 | Jamaica | | | Quantitative | | Emergency Severity Index (ESI) | Physicians and nurses (specifically those selected as trainers) working in 19 public hospital Accident and Emergency Departments (A&Es) across four health regions in Jamaica. | Intervention: National implementation of the Emergency Severity Index (ESI) triage system using a train-the-trainer model. The intervention included pre-workshop online courses, four-day intensive workshops, and planned monthly learning collaborative webinars for maintenance. | 112 participants (57 physicians and 44 nurses) were trained and participated in the workshops. | - Implementation Process - Inner Setting - Outer Setting - Innovation - Individuals | Barriers   - Poor contextualization   Facilitators   - Simple and user-friendly tools - Structured implementation planning and readiness - Contextual and cultural adaptability - National legislations and regulatory framework - Stakeholder engagement and partnership - Capacity building and training - Robust implementation support structure - Leadership commitment - Continuous M&E and Quality Improvement - Sustainable financing | Medium Risk |
| Implementing the Emergency Triage, Assessment and Treatment plus admission care (ETAT+) clinical practice guidelines to improve quality of hospital care in Rwandan district hospitals: healthcare workers' perspectives on relevance and challenges | Hategeka et al, 2017 | | | Rwanda | | Qualitative | ETAT+ | Healthcare workers (HCWs), specifically nurses, midwives, and physicians, working in district hospitals in Rwanda who were enrolled in the ETAT+ training program. | Exposure: ETAT+ clinical practice guidelines and training were being rolled out nationally, independent of this study. The study explored healthcare workers' perspectives on the relevance and implementation challenges of this external rollout. | 171 healthcare workers (who returned complete questionnaires from an initial enrollment of 211). | - Implementation Process - Inner Setting - Outer Setting - Innovation - Individuals | Barriers   - Complex, deficient and unclear triage tools and protocols - Lack of policy support - Low staff motivation and engagement - Unsupportive work environment - Unstable staffing structure - Inadequate resources and equipment - Poor implementation planning - Insufficient training and mentorship - Tecnology limitations   Facilitators   - Clear protocols and processes - Structured implementation planning and readiness - Contextual and cultural adaptability - National legislations and regulatory framework - Stakeholder engagement and partnership - Positive staff perception and attitude - Capacity building and training - Leadership commitment - Supporting staffing and supervision | Medium Risk |
| Improving care pathways for children with severe illness through implementation of the ASPIRE mHealth primary ETAT package in Malawi | Desmond et al, 2024 | Malawi-Balntyre district | | | Mixed Methods | | mHealth primary ETAT algorithm (based on ETAT) | Children (aged 0 to 14 years) presenting at primary health centres and Healthcare Workers (HCWs). | Intervention: Implementation of the ASPIRE mHealth primary ETAT package | 209,174 children were recorded by the system (with 155,931 having complete triage data); 877 healthcare workers were trained; and 70 semi-structured interviews (37 providers and 33 users) were conducted for the qualitative evaluation | - Implementation Process - Inner Setting - Outer Setting - Innovation - Individuals | Barriers   - Poor System Integration / Fragmented System (Across Levels of Care)   Facilitators   - Integrated teamwork practice - Stakeholder engagement and partnership - Capacity building and training - Efficient layout and infrastructure - Robust implementation support structure - Leadership commitment - Availability of resources—meds, equipment, and diagnostics - Standardized documentation and reporting - Continuous M&E and Quality Improvement - Community engagement and awareness - Digital and e-triage systems - Visual triage aids | Low Risk |
| Improving pediatric care in Uganda with a digital platform and quality improvement initiative: A retrospective review of Smart Triage + QI | Goertzen et al, 2025 | | | Uganda | | Quantitative | Smart Triage risk prediction algorithm and digital platform | Children up to 17 years of age presenting with acute care needs to the outpatient department (OPD) (excluding patients presenting for vaccinations, elective surgery, or wound dressing changes), and multidisciplinary OPD clinical staff (doctors, intern doctors, registered nurses, clinical officers, laboratory technicians, records staff, department heads) and non-clinical personnel (security guards), at Holy Innocents Children’s Hospital  (HICH), a dedicated pediatric hospital in Mbarara, Uganda | Intervention: Implementation of the Smart Triage digital platform combined with a continuous Quality Improvement (QI) program to optimize outpatient triaging rates and reduce time-to-antimicrobials. The intervention components consisted of a Digital Platform and a QI initiative including: Standardize Training, Workflow adjustment, and QI Team Communication. | 1 facility (121,521 pediatric patients tracked; 29 staff members trained). | - Implementation Process - Inner Setting - Innovation - Individuals | Facilitators   - Clear protocols and processes - Integrated teamwork practice - Capacity building and training - Efficient layout and infrastructure - Robust implementation support structure - Leadership commitment - Supporting staffing and supervision - Standardized documentation and reporting - Continuous M&E and Quality Improvement - Digital and e-triage systems - Safe environment - Visual triage aids | Medium Risk |
| Lessons from the frontline: The value of emergency care processes and data to pandemic responses across the Pacific region | Mitchell et al, 2022 | Fiji, Kiribati, Palau, Papua New Guinea, Samoa, Solomon Islands, Timor Leste, Tonga and Vanuatu, Micronesia (Federated States of Micronesia, Kiribati, Marshall Islands, Nauru, Palau and the northern Pacific states), Polynesia (Cook Islands, Samoa, Tokelau, Tonga, Tuvalu and other Small Island states) and Melanesia (Fiji, Papua New Guinea, Solomon Islands and Vanuatu, as well as Timor Leste) | | | Qualitative | | N/A | Emergency care clinicians and other relevant stakeholders from more than 14 Pacific Island Countries and Territories (PICTs). | Exposure: A multi-country qualitative reflection on existing emergency care processes and data systems during the COVID-19 pandemic; no single intervention was delivered. | 116 participants. | - Implementation Process - Inner Setting - Outer Setting - Innovation - Individuals | Barriers   - Poor Integration Within the Hospital - Poor System Integration / Fragmented System (Across Levels of Care) - Limited leadership commitment - Inadequate surge planning   Facilitators   - Simple and user-friendly tools - Clear protocols and processes - Effective referral and transport | Medium Risk |
| Midwives practice challenges in triaging expectant mothers: a qualitative study in Mpumalanga maternity units, South Africa | Ngwenya et al, 2025 | | | South Africa | | Qualitative | N/A | Registered midwives with more than 1 year of experience in providing midwifery care services and Expectant mothers (pregnant women) presenting to maternity units for clinical evaluation at two district hospitals located in the Ehlanzeni and Nkangala districts of Mpumalanga province, South Africa. | Exposure: No standardized triage protocol was described. The study explored midwives' practice challenges in triaging expectant mothers. | 2 facilities (20 midwives interviewed) | - Inner Setting - Innovation - Individuals | Barriers   - Complex, deficient and unclear triage tools and protocols - Poor Integration Within the Hospital - Community mistrust and poor communication - Low staff motivation and engagement - Inefficient infrastructure and patient flow - Poor teamwork and task-sharing - Unstable staffing structure - Inadequate resources and equipment - Limited leadership commitment | Low Risk |
| Multimodal learning for emergency department triage implementation: experiences from Papua New Guinea during the COVID-19 pandemic | Mitchell et al, 2023 | Papua New Guinea | | | Mixed Methods | | IITT | Emergency Department (ED) staff, including doctors, nurses, health extension officers, community health workers, and administrators or clerks. | Intervention: A multimodal training program, primarily delivered through a digital learning platform accessible via smartphone, supplemented by in-person mentoring and review tutorials from specialist emergency nurses. | 136 eligible ED staff, with 114 commencing the training, 91 completing the full program, and 30 completing the evaluation survey. | - Implementation Process - Inner Setting - Innovation - Individuals | Barriers   - Inefficient infrastructure and patient flow - Poor hospital processes - Unstable staffing structure - Inadequate resources and equipment - Insufficient training and mentorship - Tecnology limitations   Facilitators   - Structured implementation planning and readiness - Stakeholder engagement and partnership - Capacity building and training - Efficient layout and infrastructure - Robust implementation support structure - Digital and e-triage systems - Visual triage aids | Low Risk |
| Nurse-led implementation of ETAT+ is associated with reduced mortality in a children's hospital in Freetown, Sierra Leone | Hands et al, 2020 | | | Sierra Leone | | Quantitative | N/A | Outpatient department clinical staff and pediatric patients presenting to the outpatient department of Ola During Children’s Hospital (the tertiary paediatric hospital for Sierra Leone) in Freetown, Sierra Leone. | Intervention: A quality improvement and task-sharing triage program utilizing the (ETAT/ETAT+) framework. The intervention consisted ofEducational Training; Mentorship & Support; Facility and layout improvements; Extended Nurse Scope (Task-Sharing); and Operational Monitoring. | NA | - Implementation Process - Inner Setting - Individuals | Facilitators   - Positive staff perception and attitude - Capacity building and training - Efficient layout and infrastructure - Robust implementation support structure - Leadership commitment - Supporting staffing and supervision - Availability of resources—meds, equipment, and diagnostics | Medium Risk |
| Nurses' and doctors' perceptions regarding the implementation of a triage system in an emergency unit in South Africa | Augustyn et al, 2009 | South Africa | | | Quantitative | | Cape Triage Score (CTS) | All nurses and doctors involved  in receiving patients in the emergency unit | Exposure: The Cape Triage Score (CTS) was implemented at the study site independently of this research. This cross-sectional, post-only survey measured nurses' and doctors' perceptions after that implementation; no baseline data were collected and no intervention was delivered by the study team. | 1 facility (15 healthcare professionals surveyed; nurses and doctors). | - Implementation Process - Inner Setting - Innovation - Individuals | Barriers   - Poor hospital processes - Inadequate resources and equipment - Insufficient training and mentorship   Facilitators   - Simple and user-friendly tools - Positive staff perception and attitude - Capacity building and training - Efficient layout and infrastructure - Leadership commitment - Supporting staffing and supervision | Medium Risk |
| Nurses' Perception on the Hindrances of Triage System in Emergency Unit | Olofinbiyi et al, 2024 | | | South Africa | | Quantitative | SATS | Nurses working in the Emergency Department (ED), Pediatric Unit (PU), and Outpatient Department (OPD) at a selected public hospital in KwaZulu-Natal, South Africa | Exposure: SATS was already in use at the study site. The study examined nurses' perceptions of the existing system; no new intervention was delivered. | 100 nurses | - Implementation Process - Inner Setting - Innovation - Individuals | Barriers   - Complex, deficient and unclear triage tools and protocols - Community mistrust and poor communication - Inefficient infrastructure and patient flow - Unsupportive work environment - Poor teamwork and task-sharing - Unstable staffing structure - Low staff confidence and buy-in - Inadequate resources and equipment - Insufficient training and mentorship | Low Risk |
| Obstetric triage improvement process using the Donabedian model of quality care: a quality improvement initiative | Naz et al. 2022 | Pakistan | | | Quantitative | | Triage four-tiered acuity tool for obstetric triage (Obstetric Acuity Tool) | Pregnant women presenting with pregnancy-related or non-obstetric complaints to the labour and delivery (L&D) triage area of a tertiary care hospital in Karachi, Pakistan. | Intervention: Revamping of the obstetric triage system including: structural changes to create designated assessment areas; implementation of a four-tiered obstetric triage acuity tool; introduction of new triage guidelines and flow processes; and specialized staff training with a dedicated staffing model. | 200 women (a convenience sample consisting of 100 participants in the pre-intervention audit and 100 in the post-implementation audit). | - Implementation Process - Inner Setting - Innovation - Individuals | Facilitators   - Simple and user-friendly tools - Clear protocols and processes - Structured implementation planning and readiness - Contextual and cultural adaptability - Integrated teamwork practice - Capacity building and training - Efficient layout and infrastructure - Robust implementation support structure - Leadership commitment - Supporting staffing and supervision - Availability of resources—meds, equipment, and diagnostics - Standardized documentation and reporting - Continuous M&E and Quality Improvement - Visual triage aidsSimple and user-friendly tools - Clear protocols and processes - Structured implementation planning and readiness - Contextual and cultural adaptability - Integrated teamwork practice - Capacity building and training - Efficient layout and infrastructure - Robust implementation support structure - Leadership commitment - Supporting staffing and supervision - Availability of resources—meds, equipment, and diagnostics - Standardized documentation and reporting - Continuous M&E and Quality Improvement - Visual triage aidsSimple and user-friendly tools - Clear protocols and processes - Structured implementation planning and readiness - Contextual and cultural adaptability - Integrated teamwork practice - Capacity building and training - Efficient layout and infrastructure - Robust implementation support structure - Leadership commitment - Supporting staffing and supervision - Availability of resources—meds, equipment, and diagnostics - Standardized documentation and reporting - Continuous M&E and Quality Improvement - Visual triage aidsSimple and user-friendly tools - Clear protocols and processes - Structured implementation planning and readiness - Contextual and cultural adaptability - Integrated teamwork practice - Capacity building and training - Efficient layout and infrastructure - Robust implementation support structure - Leadership commitment - Supporting staffing and supervision - Availability of resources—meds, equipment, and diagnostics - Standardized documentation and reporting - Continuous M&E and Quality Improvement - Visual triage aidsSimple and user-friendly tools - Clear protocols and processes - Structured implementation planning and readiness - Contextual and cultural adaptability - Integrated teamwork practice - Capacity building and training - Efficient layout and infrastructure - Robust implementation support structure - Leadership commitment - Supporting staffing and supervision - Availability of resources—meds, equipment, and diagnostics - Standardized documentation and reporting - Continuous M&E and Quality Improvement - Visual triage aids | Medium Risk |
| Paediatric emergency triage, assessment and treatment (ETAT) - preparedness for implementation at primary care facilities in Malawi | King et al, 2021 | | | Malawi | | Mixed Methods | ETAT | Healthcare providers (including clinical officers, medical assistants, nurses, midwives, health surveillance assistants, and attendants) and frontline health facilities (dispensaries, primary health centres, rural and community hospitals) in the Zomba and Mchinji districts of Malawi. | Exposure: The study assessed facility readiness/preparedness for future ETAT implementation; no intervention was delivered during the study period. | 47 facilities, 531 healthcare providers, 6 focus group discussions, and 5 semi-structured interviews. | - Implementation Process - Inner Setting - Outer Setting - Innovation - Individuals | Barriers   - Complex, deficient and unclear triage tools and protocols - Poor System Integration / Fragmented System (Across Levels of Care) - Community mistrust and poor communication - Low staff motivation and engagement - Inefficient infrastructure and patient flow - Unsupportive work environment - Unstable staffing structure - Inadequate resources and equipment - Insufficient training and mentorship - Weak monitoring and evaluation   Facilitators   - Stakeholder engagement and partnership - Positive staff perception and attitude - Robust implementation support structure - Supporting staffing and supervision | Low Risk |
| Perceptions of doctors and nurses at a Ugandan hospital regarding the introduction and use of the South African Triage Scale | Mulindwa et al, 2016 | Uganda | | | Qualitative | | SATS | All fulltime-employed doctors and nurses working in the International Hospital Kampala (IHK) OPD and EU with work experience of between 1 and 6 years. | Intervention: SATS was introduced and implemented by the researcher at IHK. Two initial one-hour training sessions were held for OPD/EU nurses and doctors, plus a separate session for doctors in other departments; a clinical audit was conducted after 5 weeks of use, followed by a follow-up training session to improve consistency. Perceptions were collected via in-depth interviews after 6 months of continuous SATS use. | 1 facility (16 clinical staff interviewed: 12 nurses, 4 doctors). | - Implementation Process - Inner Setting - Innovation - Individuals | Barriers   - Complex, deficient and unclear triage tools and protocols - Low staff motivation and engagement - Unstable staffing structure - Low staff confidence and buy-in - Inadequate resources and equipment   Facilitators   - Simple and user-friendly tools - Contextual and cultural adaptability - Integrated teamwork practice - Positive staff perception and attitude - Capacity building and training - Efficient layout and infrastructure - Leadership commitment - Visual triage aids | Low Risk |
| Practice of paediatric triage among nurses in human-resource constrained setting: A cross-sectional study in the Tamale metropolis of Ghana | Pwavra et al, 2022 | | | Ghana | | Quantitative | N/A | Nurses (including enrolled, diploma, and degree-holding nurses) working at the Paediatric Units/Emergency Departments of three public hospitals (Tamale Teaching Hospital, Tamale West Hospital, and Tamale Central Hospital) in Tamale, Ghana. | Exposure: No standardized triage tool was described. The study assessed nurses' paediatric triage practices in a resource-constrained setting. | 173 nurses (successfully completed questionnaires representing a 97.7% response rate from an original sample size of 177). | - Implementation Process - Inner Setting - Innovation | Barriers   - Complex, deficient and unclear triage tools and protocols - Unstable staffing structure - Inadequate resources and equipment - Insufficient training and mentorship | Low Risk |
| Public hospital management in times of crisis: Lessons learned from Cienfuegos, Cuba (1996-2008) | De Vos et al, 2010 | Cuba | | | Editorial | | N/A | Healthcare staff and patients at the General University Hospital Dr. Gustavo Aldereguia Lima in Cienfuegos, Cuba. | Exposure: A retrospective case study of hospital reforms (1996-2008), including introduction of a color-coded triage system, reconstructed from hospital reports and interviews. Not a prospective intervention trial; no new intervention was delivered as part of this study. | The study analyzes data from the entire hospital, which handles approximately 22,000 hospitalizations per year. Qualitative data was derived from interviews with key decision-makers and a participatory group who designed the evaluation tools. | - Implementation Process - Inner Setting - Outer Setting - Innovation - Individuals | Facilitators   - Simple and user-friendly tools - Clear protocols and processes - Integrated teamwork practice - Stakeholder engagement and partnership - Positive staff perception and attitude - Capacity building and training - Efficient layout and infrastructure - Leadership commitment - Supporting staffing and supervision - Continuous M&E and Quality Improvement - Community engagement and awareness | Low Risk |
| Quality and effectiveness of a pediatric triage training program in a Guatemalan public hospital | Crouse et al, 2016 | | | Guatemala | | Quantitative | ETAT (Spanish version: CETEP) | Pediatric health care workers (including physicians, residents, senior medical students, nurses, and firemen) at Hospital Nacional Pedro Bethancourt (HNPB), a secondary-level public hospital in Guatemala. | Intervention: Development and implementation of a pediatric Emergency Triage Assessment and Treatment (ETAT) training program in Spanish “Clasificación, Evaluación y Tratamiento de Emergencias Pediátricas (CETEP). The intervention included a train-the-trainer course followed by local trainings. | 249 health care workers were trained by 24 facilitators. Additionally, 285 patients were used to assess the accuracy of triage category assignments. | - Implementation Process - Innovation - Individuals | Facilitators   - Contextual and cultural adaptability - Stakeholder engagement and partnership - Capacity building and training - Leadership commitment | Low Risk |
| Responsibility-evading performance: the experiences of healthcare staff about triage in emergency departments: a qualitative study | Sherafat et al, 2019 | Iran | | | Qualitative | | N/A | Healthcare staff with at least two years of triage-related experience, including triage nurses, emergency general physicians, emergency medicine specialists, and expert managers in hospitals and educational/administrative centres in Yazd, Iran. | Exposure: No standardized triage tool was described. The study explored healthcare staff experiences with existing triage practices. | 25 participants (who participated in a total of 30 interviews). | - Implementation Process - Inner Setting - Innovation - Individuals | Barriers   - Complex, deficient and unclear triage tools and protocols - Unstable staffing structure - Low staff confidence and buy-in - Weak monitoring and evaluation   Facilitators   - Capacity building and training | Low Risk |
| Strategies to enhance knowledge and practical skills of triage amongst nurses working in the emergency departments of rural hospitals in South Africa. | Phukubye et al, 2021 | | | South Africa | | Qualitative | N/A | Emergency nurses (including registered/professional nurses and specialty trained nurses) working in the emergency departments of rural hospitals in the Sekhukhune District of Limpopo Province, South Africa | Exposure: No standardized triage tool was described. The study examined strategies to enhance nurses' existing triage knowledge and skills. | 17 professional nurses | - Implementation Process - Individuals | Facilitators   - Clear protocols and processes - Capacity building and training - Leadership commitment - Supporting staffing and supervision - Continuous M&E and Quality Improvement | Low Risk |
| Strengthening emergency care systems to mitigate public health challenges arising from influxes of Iidividuals with different socio-cultural backgrounds to a level one emergency center in South East Europe | Twomey et al, 2018 | Serbia | | | Editorial | | Modified version of SATS | Emergency department staff and patients at the Clinical Centre of Serbia (CCS) in Belgrade. This includes the general population of Serbia as well as an influx of people seeking asylum or in transit. | Intervention: The introduction and integration of a standardized triage process into the acute care pathways at the Clinical Centre of Serbia (CCS). | NA | - Implementation Process - Inner Setting - Innovation - Individuals | Barriers   - Inefficient infrastructure and patient flow - Unstable staffing structure - Inadequate resources and equipment - Inadequate documentation - Insufficient training and mentorship   Facilitators   - Simple and user-friendly tools - Structured implementation planning and readiness - Contextual and cultural adaptability - Capacity building and training - Efficient layout and infrastructure - Robust implementation support structure - Leadership commitment - Supporting staffing and supervision - Availability of resources—meds, equipment, and diagnostics - Standardized documentation and reporting - Continuous M&E and Quality Improvement - Digital and e-triage systems | Low Risk |
| Studying the efficiency triage at Shahid Beheshti Hospitals, Tehran, Iran | Derakhshanfar et al, 2015 | | | Iran | | Quantitative | ESI Version 4 | Children below the age of 14 who referred to the emergency departments of hospitals affiliated with Shahid Beheshti University of Medical Sciences in Tehran, Iran. | Exposure: ESI (Version 4) was already in use at the study site. The study evaluated triage efficiency under the existing system; no new intervention was delivered. | 1,453 patient files. | - Implementation Process - Inner Setting - Individuals | Barriers   - Inefficient infrastructure and patient flow - Insufficient training and mentorship - Limited stakeholders engagement - Weak monitoring and evaluation | Low Risk |
| Sudanese emergency departments: a study to identify the barriers to a well-functioning triage | Ibrahim, 2022 | Sudan | | | Quantitative | | Sudan Triage Tool, IITT, hospital based triage | Emergency department staff, including doctors (house officers, medical officers, registrars, specialists, consultants), nurses, matrons, and receptionists, working in eight public hospitals in Khartoum State, Sudan. | Exposure: No formal triage system was in place in Khartoum hospitals at the time of this cross-sectional study ("Currently there is no formal triage system in the State of Khartoum and has yet to be established"). The study surveyed ED staff to identify barriers to establishing a well-functioning triage system; no new intervention was delivered. | 185 participants (from an initial calculated sample size of 200, representing a 92.5% response rate). | - Implementation Process - Inner Setting - Outer Setting - Innovation - Individuals | Barriers   - Complex, deficient and unclear triage tools and protocols - Inadequate resources and equipment - Inadequate documentation - Insufficient training and mentorship - Inadequate safety protocols   Facilitators   - Structured implementation planning and readiness - National legislations and regulatory framework - Capacity building and training - Leadership commitment - Availability of resources—meds, equipment, and diagnostics - Sustainable financing | Medium Risk |
| The effectiveness of the South African Triage Score (SATS) in a rural emergency department | Rosedale et al, 2011 | | | KwaZulu-Natal | | Quantitative | SATS | Patients attending the emergency department (ED) of Ngwelezane Hospital, a government referral hospital in rural KwaZulu-Natal, South Africa. The study excluded children under 12, patients who were "seen and treated" without being formally triaged, and those admitted directly to resuscitation or high-dependency units. | Exposure: SATS was already implemented at the study site. The study compared its diagnostic accuracy against Modified Early Warning Score (MEWS); no new intervention was delivered. | 589 patients (from an initial 640 participants, 92% had complete datasets for analysis). | - Inner Setting | Barriers   - Unstable staffing structure | Medium Risk |
| The impact of the clinical audit cycle on the implementation of hospital triage in the emergency department | Taban et al. 2024 | Iran | | | Quantitative | | ESI | ED nurses (at Golestan Hospital in Ahvaz, southwest Iran. | Intervention: Implementation of a 6-stage clinical audit cycle to evaluate and improve nurse accuracy using ESI triage algorithm | 1 facility (770 triage events audited; 8 nurses trained) | - Implementation Process - Individuals | Facilitators   - Capacity building and training - Robust implementation support structure - Leadership commitment - Continuous M&E and Quality Improvement | Medium Risk |
| The introduction of a midwife-led obstetric triage system into a regional referral hospital in Ghana | Floyd et al, 2018 | | | Ghana | | Quantitative | Locally developed obstetric triage protocol with wristbands and a one-page triage assessment form | Midwives and medical staff working on the labour ward, theatre, and antenatal/postnatal wards at Ridge Regional Hospital in Accra, Ghana. The study also evaluated outcomes for pregnant women presenting to the labour ward. | Intervention: The introduction of a midwife-led obstetric triage system, which included: an interactive triage training course; the use of coloured risk acuity wristbands (red, yellow, green); and the implementation of a standardized one-page triage assessment form. | 62 midwives received the triage training. Evaluation components included feedback from 25 staff members, an audit of 535 wristbands, and a review of 203 admissions for waiting time analysis. | - Implementation Process - Inner Setting - Innovation - Individuals | Barriers   - Poor Integration Within the Hospital - Poor system integration/fragmented system - Inefficient infrastructure and patient flow - Poor hospital processes - Inadequate resources and equipment - Inadequate documentation - Weak monitoring and evaluation   Facilitators   - Clear protocols and processes - Structured implementation planning and readiness - Contextual and cultural adaptability - Stakeholder engagement and partnership - Capacity building and training - Efficient layout and infrastructure - Robust implementation support structure - Leadership commitment - Supporting staffing and supervision - Standardized documentation and reporting - Continuous M&E and Quality Improvement - Visual triage aids | High Risk |
| Triage implementation in resource-limited emergency departments: sharing tools and experience from the Pacific region | Mitchell et al, 2024 | Vanuatu, a Pacific Small Island Developing State | | | Editorial | | IITT | Healthcare workers and patients at Vila Central Hospital (VCH) in Port Vila, Vanuatu, as well as the broader emergency department systems in Pacific Island Countries and Territories (PICTs) | Intervention: Implementation of the WHO Interagency Integrated Triage Tool (IITT) supported by four key strategies: needs assessment, digital learning, public communications, and electronic data management. | N/A (Implementation case study of 1 emergency department). | - Implementation Process - Inner Setting - Outer Setting - Individuals | Barriers   - Inadequate documentation - Insufficient training and mentorship   Facilitators   - Clear protocols and processes - Structured implementation planning and readiness - Integrated teamwork practice - Stakeholder engagement and partnership - Effective referral and transport - Capacity building and training - Efficient layout and infrastructure - Leadership commitment - Supporting staffing and supervision - Standardized documentation and reporting - Continuous M&E and Quality Improvement - Community engagement and awareness - Digital and e-triage systems | Medium Risk |
| Triage practices for emergency care delivery: a qualitative study among febrile patients and healthcare workers in a tertiary care hospital in Nepal | Adhikari et al, 2024 | | | Nepal | | Qualitative | WHO color-coded triage system (Red, Yellow, Green) | Febrile patients and ED healthcare providers including nurse-in-charge, medical officers, a specialist in emergency medicine, and a health assistant, at at the ED of Tribhuvan University Teaching Hospital (TUTH), Nepal. | Exposure: The WHO color-coded triage system was already in use at the study site. The study explored triage practices for febrile patients and healthcare workers; no new intervention was delivered. | 1 facility (15 patients interviewed; 15 healthcare workers interviewed; 20 direct clinical observation sessions) | - Implementation Process - Inner Setting - Outer Setting - Individuals | Barriers   - Community mistrust and poor communication - Inefficient infrastructure and patient flow - Unsupportive work environment - Poor teamwork and task-sharing - Poor hospital processes - Unstable staffing structure - Inadequate resources and equipment - Insufficient training and mentorship   Facilitators   - Clear protocols and processes - Integrated teamwork practice - Effective referral and transport - Positive staff perception and attitude - Efficient layout and infrastructure | Low Risk |
| Triage processes in primary, secondary, and tertiary health care facilities in the Kathmandu Valley, Nepal: a mixed-methods study | Shrestha et al, 2024 | Nepal | | | Mixed Methods | | N/A | Adult patients (for interviews and observations) and healthcare workers (HCWs), including medical officers, staff nurses, and health assistants, across three tiers of health facilities (primary care centers (PHC; n = 6), secondary care centers (SHC; n = 3), and tertiary care hospital (n = 1)) | Exposure: No single standardized triage tool was described. The study examined triage processes across primary, secondary, and tertiary facilities. | 30 patients for qualitative interviews and observations; 144 healthcare workers for the quantitative survey. | - Implementation Process - Inner Setting - Outer Setting - Innovation - Individuals | Barriers   - Complex, deficient and unclear triage tools and protocols - Community mistrust and poor communication - Inefficient infrastructure and patient flow - Unsupportive work environment - Unstable staffing structure - Low staff confidence and buy-in - Inadequate resources and equipment - Insufficient training and mentorship   Facilitators   - Clear protocols and processes - Structured implementation planning and readiness - Contextual and cultural adaptability - Positive staff perception and attitude - Capacity building and training - Efficient layout and infrastructure - Robust implementation support structure - Leadership commitment - Availability of resources—meds, equipment, and diagnostics - Community engagement and awareness - Digital and e-triage systems | Low Risk |
| Triaging the triage: reducing waiting time to triage in the emergency department at a tertiary care hospital in New Delhi, India | Kumar et al, 2019 | | | India | | Quantitative | Simple red/yellow/green classification | Patients attending the general adult Emergency Department (ED) and multidisciplinary triage team (including resident doctors, nursing officers, health assistants, and security guards) at a tertiary care hospital in New Delhi, India. | Intervention: A multifaceted quality improvement initiative focused on reducing triage waiting times. Key components included:Multidisciplinary QI team formation; Staff orientation and refresher training on red triage protocols; A formal first responder course for non-physician staff; Providing extra EM consultant support during peak hours; Reducing staff turnover through fixed-term rosters; Streamlining documentation (Introducing preprinted triage registers and removing duplicate entry); Using color-coded wristbands; and Changing the arrangement of the triage room. | For measurement purposes, data were collected for 20 patients per day, twice a week. The broader context involved a facility seeing over 400 patients daily, with monthly census ranging from 8,936 to over 15,590 patients during the study period. | - Implementation Process - Inner Setting - Innovation - Individuals | Barriers   - Lack of policy support - Poor Integration Within the Hospital - Inefficient infrastructure and patient flow - Poor teamwork and task-sharing - Unstable staffing structure - Inadequate resources and equipment - Inadequate documentation - Insufficient training and mentorship   Facilitators   - Integrated teamwork practice - Stakeholder engagement and partnership - Capacity building and training - Robust implementation support structure - Leadership commitment - Supporting staffing and supervision - Standardized documentation and reporting - Continuous M&E and Quality Improvement - Visual triage aids | Low Risk |
| User embracement with risk rating: assessment of dimension outcome in the professionals’ perspective | Versa et al, 2016 | Brazil | | | Quantitative | | User Embracement with Risk Rating (ACCR) | Healthcare and support professionals (including nurses, doctors, social workers, and operational, administrative, and security agents) working in Emergency Hospital Services (EHS) at four units in the states of Paraná and São Paulo, Brazil. | Exposure: ACCR system was already in use across the four Emergency Hospital Services studied; no new intervention was delivered. | 314 subjects. | - Inner Setting - Outer Setting - Innovation - Individuals | Barriers   - Complex, deficient and unclear triage tools and protocols - Poor System Integration / Fragmented System (Across Levels of Care) - Poor system integration/fragmented system - Community mistrust and poor communication - Low staff motivation and engagement - Inefficient infrastructure and patient flow - Unsupportive work environment - Unstable staffing structure - Inadequate resources and equipment - Limited leadership commitment   Facilitators   - Simple and user-friendly tools - Effective referral and transport - Positive staff perception and attitude - Supporting staffing and supervision | Medium Risk |

**Abbreviations:** ETAT = Emergency Triage, Assessment and Treatment; IITT = Interagency Integrated Triage Tool; SATS = South African Triage Scale; ATS = Australian Triage Scale; ESI = Emergency Severity Index.

***Note.*** "Intervention" indicates an activity, tool, or program actively delivered or implemented as part of the study (n = 29). "Exposure" indicates a pre-existing triage system or practice already in place, which the study describes, evaluates, or surveys perceptions of without introducing something new (n = 28).
