# Supplementary material for: Identifying barriers and facilitators to implementing triage systems in low- and middle-income countries: A scoping review
Source: PLOS Glob Public Health. 2026 Sep 29;6(9):e0007378. doi: 10.1371/journal.pgph.0007378 (PMC13623090; doi:10.1371/journal.pgph.0007378)
Supplement: Supplementary file 5 [file pgph.0007378.s004.docx]

**S3 Table. Quality Appraisal**

I. **Qualitative Studies - JBI**

| **Title** | **Year of publication** | **Country(s)** | **Study design** | **Method aligns with question** | **Method aligns with data collection** | **Method aligns with analysis** | **Method aligns with interpretation** | **Researcher role stated** | **Researcher influence addressed** | **Participants adequately represented** | **Ethics approval** | **Findings supported by data** | **Conclusions flow from data** | **Risk of Bias** |
| --- | --- | --- | --- | --- | --- | --- | --- | --- | --- | --- | --- | --- | --- | --- |
| Responsibility-Evading Performance: The Experiences of Healthcare Staff about Triage in Emergency Departments: A Qualitative Study | 2019 | Iran | Qualitative | Y | Y | Y | Y | Partially | N | Y | Y | Y | Y | Low |
| Triage practices for emergency care delivery: a qualitative study among febrile patients and healthcare workers in a tertiary care hospital in Nepal | 2024 | Nepal | Qualitative | Y | Y | Y | Y | N | N | Y | Y | Y | Y | Low |
| Factors influencing the implementation of obstetric triage: a qualitative study | 2021 | Iran | Qualitative | Y | Y | Y | Y | Y | N | Y | Y | Y | N | Low |
| Health worker perspectives of Smart Triage, a digital triaging platform for quality improvement at a referral hospital in Uganda: a qualitative analysis | 2022 | Uganda | Qualitative | Y | Y | Y | Y | Y | Y | Y | Y | Y | Y | Low |
| Explaining challenges of obstetric triage structure: A qualitative study | 2020 | Iran | Qualitative | Y | Y | Y | Y | N | N | Y | Y | Y | Y | Low |
| Assessment of Attitudes Toward the Emergency Triage System in Belize | 2020 | Belize | Qualitative | Y | Y | Y | Y | N | N | Y | Y | Y | Y | Low |
| Implementing the Emergency Triage, Assessment and Treatment plus admission care (ETAT+) clinical practice guidelines to improve quality of hospital care in Rwandan district hospitals: healthcare workers' perspectives on relevance and challenges. | 2017 | Rwanda | Qualitative | Y | N | Y | Y | N | N | Y | Y | Y | Y | Medium |
| Lessons from the frontline: The value of emergency care processes and data to pandemic responses across the Pacific region | 2022 | 14 PICTs | Qualitative | Y | Y | Y | Y | N | N | N | Y | Y | Y | Medium |
| Perceptions of doctors and nurses at a Ugandan hospital regarding the introduction and use of the South African Triage Scale | 2016 | Uganda | Qualitative | Y | Y | Y | Y | Y | N | Y | Y | Y | N | Low |
| Strategies to Enhance Knowledge and Practical Skills of Triage amongst Nurses Working in the Emergency Departments of Rural Hospitals in South Africa | 2021 | South Africa | Qualitative | Y | Y | Y | Y | N | N | Y | Y | Y | Y | Low |
| Adapting a novel emergency triage tool to a resource-limited hospital in Nepal | 2025 | Nepal | Qualitative | Y | Y | Y | Y | N | N | N | Y | Y | Y | Medium |
| Midwives' practice challenges in triaging expectant mothers: a qualitative study in Mpumalanga maternity units, South Africa | 2025 | South Africa | Qualitative | Y | Y | Y | Y | Y | Y | Y | Y | Y | Y | Low |

III. **Cross-sectional studies - JBI**

| **Title** | **Year of publication** | **Country(s)** | **Study design** | **Inclusion criteria clear** | **Subjects/setting described** | **Exposure measured validly** | **Standard criteria for condition** | **Confounders identified** | **Confounding strategies stated** | **Outcomes measured validly** | **Appropriate analysis** | **Risk of Bias** |
| --- | --- | --- | --- | --- | --- | --- | --- | --- | --- | --- | --- | --- |
| Assessment of hospital-based adult triage at emergency receiving areas in hospitals in Northern Uganda. | 2017 | Uganda | Descriptive cross-sectional | Y | Y | Y | Y | N | N | Y | Y | Medium |
| Nurses’ Perception on the Hindrances of Triage System in Emergency Unit | 2024 | South Africa | Descriptive cross-sectional | Y | Y | Y | Y | N | N | Y | Y | Medium |
| A quality improvement study of the emergency centre triage in a tertiary teaching hospital in northern Ethiopia | 2017 | Ethiopia | Cross-sectional quality improvement audit | Y | Y | Y | Y | N | N | Y | Y | Medium |
| Assessment of knowledge and skills of triage amongst nurses working in the emergency centres in Dar es Salaam, Tanzania | 2014 | Tanzania | Cross-sectional quality improvement audit | Y | Y | Y | Y | Y | N | Y | Y | Medium |
| Nurses' and doctors perceptions regarding the implementation of a triage system in an emergency unit in South Africa | 2009 | South Africa | Cross-sectional quantitative and exploratory | Y | Y | Y | Y | Y | N | Y | Y | Medium |
| Implementation of a triage score system in an emergency room in Timergara, Pakistan | 2013 | Pakistan | Cross-sectional | N | Y | Y | Y | N | N | Y | Y | Medium |
| User embracement with risk rating: assessment of dimension "outcome" in the professional's perspective | 2016 | Brazil | Cross-sectional | Y | Y | Y | Y | Y | N | Y | Y | Medium |
| Emergency nurses’ knowledge and experience with the triage process in Hunan Province, China | 2017 | China | Cross-sectional | Y | Y | Y | Y | Y | Y | Y | Y | Low |
| Sudanese emergency departments: a study to identify the barriers to a well-functioning triage | 2022 | Sudan | Cross-sectional | Y | Y | Y | Y | Y | N | Y | Y | Medium |
| Practice of paediatric triage among nurses in human-resource constrained setting: A cross-sectional study in the Tamale metropolis of Ghana | 2023 | Ghana | Cross-sectional | Y | Y | Y | Y | Y | Y | Y | Y | Low |
| The Effectiveness of the South African Triage Score (SATS) in a Rural Emergency Department | 2011 | South Africa | Cross-sectional | Y | Y | Y | Y | Y | N | Y | Y | Medium |
| Studying the efficiency triage at Shahid Beheshti Hospitals, Tehran, Iran | 2015 | Iran | Cross-sectional | N | N | Y | Y | Y | Y | Y | Y | Low |
| Effect of Emergency Nurses' Work Stress, Workload, and Motivation on Emergency Severity Index Implementation | 2024 | Iran | Cross-sectional | Y | N | Y | Y | Y | N | Y | Y | Medium |
| Improving pediatric care in Uganda with a digital platform and quality improvement initiative: A retrospective review of Smart Triage plus QI | 2025 | Uganda | Cross-sectional quality improvement audit | N | Y | Y | Y | Y | N | Y | Y | Medium |
| Implementing the Emergency Severity Index Triage System in Jamaican Accident and Emergency Departments. | 2019 | Jamaica | Cross-sectional | Y | Y | Y | Y | N | N | Y | Y | Medium |

V. **Mixed Methods Studies – MMAT**

| **Title** | **Year of publication** | **Country(s)** | **Study design** | **Clear research questions** | **Data sources appropriate** | **Measurements relevant** | **Integration of qualitative & quantitative results** | **Addressing divergences/limitations** | **Risk of Bias** |
| --- | --- | --- | --- | --- | --- | --- | --- | --- | --- |
| A mixed-methods study on evaluating an updated, francophone version of ETAT+ training in Madagascar | 2022 | Madagascar | Cross-sectional mixed-methods study | Y | Y | Y | Y | Y | Low |
| Multimodal learning for emergency department triage implementation: experiences from Papua New Guinea during the COVID-19 pandemic | 2023 | Papa New Guinea | Mixed Methods Study with pre- post- intervention | Y | Y | Y | N | Y | Low |
| Improving care pathways for children with severe illness through implementation of the ASPIRE mHealth primary ETAT package in Malawi. | 2024 | Malawi | Mixed methods | Y | Y | Y | N | Y | Low |
| Developing Emergency Triage Systems in Cambodia | 2020 | Cambodia | Mixed Methods Study with pre- post- intervention | Y | Y | Y | N | N | Medium |
| Paediatric Emergency Triage, Assessment and Treatment (ETAT) - preparedness for implementation at primary care facilities in Malawi. | 2021 | Malawi | Mixed Methods Study | Y | Y | Y | Y | Y | Low |
| Implementation of triage in the hospital emergency room of private hospital, Yogyakarta, Indonesia | 2015 | Indonesia | Mixed methods | Y | Y | Y | N | Y | Low |
| Triage processes in primary, secondary, and tertiary health care facilities in the Kathmandu Valley, Nepal: a mixed-methods study | 2024 | Nepal | Mixed methods | Y | Y | Y | N | Y | Low |
| Exploring the effect of implementation and context on a stepped-wedge randomised controlled trial of a vital sign triage device in routine maternity care in low-resource settings | 2019 | Ethiopia, India, Haiti, Malawi, Sierra Leone, Uganda, Zambia and Zimbabwe | Mixed Methods Study with pre- post- intervention | Y | Y | Y | Y | Y | Low |
| Implementation of the Interagency Integrated Triage Tool (IITT) in a Rural Emergency Department: A Qualitative Study in Western Nepal | 2025 | Nepal | Mixed Methods Study with pre- post- intervention | Y | Y | Y | N | Y | Low |
| Are we ready? Emergency unit capacity at selected district level hospitals in Lusaka Province, Zambia: Barriers and facilitators for improving trauma care: a mixed methods approach | 2025 | Zambia | Mixed methods | Y | Y | Y | Y | Y | Low |
| Barriers and facilitators to providing facility-based emergency care in Addis Ababa, Ethiopia: A mixed methods study | 2025 | Ethiopia | Mixed Methods | Y | Y | Y | Y | Y | Low |

VII. **Text and Opinion – JBI**

| **Title** | **Year of publication** | **Country(s)** | **Study design** | **Source identified** | **Author has standing** | **Population interests central** | **Argument logical/defended** | **Literature referenced** | **Incongruence with literature defended** | **Risk of Bias** |
| --- | --- | --- | --- | --- | --- | --- | --- | --- | --- | --- |
| Triage implementation in resource-limited emergency departments: sharing tools and experience from the Pacific region | 2024 | Vanuatu | Text/Opinion | Y | Y | Y | N/A | Y | N | Low |
| Strengthening Emergency Care Systems to Mitigate Public Health Challenges Arising from Influxes of Individuals with Different Socio-Cultural Backgrounds to a Level One Emergency Center in South East Europe | 2018 | Serbia | Protocol report | Y | Y | Y | Y | Y | N/A | Low |
| Public hospital management in times of crisis: lessons learned from Cienfuegos, Cuba (1996-2008) | 2010 | Cuba | Review, unstructured interviews | Y | Y | Y | Y | Y | N/A | Low |
| Implementation of a novel three-tier triage tool in Papua New Guinea: A model for resource-limited emergency departments | 2020 | Papa New Guinea | Health policy | Y | Y | Y | Y | Y | N/A | Low |

IX. **Quasi-Experimental – JBI**

| **Title** | **Year of publication** | **Country(s)** | **Study design** | **Cause and Effect clear?** | **Control group?** | **Participants compared are similar** | **Compared participants receive similar treatment/care, other than the exposure or intervention of interest** | **There are multiple measurements of the outcome, both pre and post the intervention/exposure** | **Outcomes measures the same way** | **Reliable outcome measurment** | **Follow-up complete and if not, differences in follow-up between groups adequately described and analyzed** | **Appropriate stat analysis** | **Risk of Bias** |
| --- | --- | --- | --- | --- | --- | --- | --- | --- | --- | --- | --- | --- | --- |
| Implementing a triage tool to improve appropriateness of care for children coming to the emergency department in a small hospital in India | 2020 | India | Quality improvement using sequential PDSA cycles | Y | N | Y | Y | Y | Y | Y | Y | Y | Low |
| Application of Queuing Theory to Optimize the Triage Process in a Tertiary Emergency Care (“ER”) Department | 2019 | Colombia | Quality improvement | Y | Y | Y | Y | Y | Y | Y | Y | Y | Low |
| Obstetric triage improvement process using the Donabedian model of quality care: a quality improvement initiative | 2022 | Pakistan | Quality improvement | Y | Y | Y | Y | N | Y | Y | N | N | Medium |
| Quality and Effectiveness of a Pediatric Triage Training Program in a Guatemalan Public Hospital | 2016 | Guatemala | Pre/post-test implementation | Y | N | Y | N/A | Y | Y | Y | N | Y | Low |
| A complex intervention to improve implementation of World Health Organization guidelines for diagnosis of severe illness in low-income settings: a quasi-experimental study from Uganda | 2017 | Uganda | Pre/post quasi experimental | Y | N | Y | N/A | Y | Y | Y | N | Y | Low |
| The introduction of a midwife-led obstetric triage system into a regional referral hospital in Ghana | 2018 | Ghana | Participatory action research | Y | N | Y | N/A | Y | Y | N | N | Y | Medium |
| Assessment of Interrater Reliability of the Emergency Severity Index After Implementation in Emergency Departments in Jamaica Using a Learning Collaborative Approach | 2021 | Jamaica | Retrospective case series review | Y | N | N | N/A | Y | Y | Y | Y | Y | Medium |
| Emergency Triage Assessment and Treatment Plus (ETAT+): adapting training to strengthen quality improvement and task-sharing in emergency paediatric care in Sierra Leone. | 2021 | Sierra Leone | Pre/post quasi experimental | Y | N | Y | N/A | Y | Y | Y | Y | Y | Low |
| Nurse-led implementation of ETAT+ is associated with reduced mortality in a children's hospital in Freetown, Sierra Leone | 2020 | Sierra Leone | Pre/post quasi experimental | Y | N | Y | N/A | Y | Y | Y | N | Y | Medium |
| Triaging the triage: reducing waiting time to triage in the emergency department at a tertiary care hospital in New Delhi, India | 2019 | India | Quality improvement using sequential PDSA cycles | Y | N | Unclear | Y | Y | Y | Y | Y | Y | Low |
| Evaluation of a digital triage platform in Uganda: A quality improvement initiative to reduce the time to antibiotic administration | 2020 | Uganda | Quality improvement with pre/post implementation | Y | N | Y | Y | Y | Y | Y | N | Y | Low |
| Capacity Building for Health Care Workers and Support Staff in Pediatric Emergency Triage Assessment and Treatment (ETAT) at Primary Health Care Level in Resource Limited Settings: Experiences from Malawi | 2022 | Malawi | Quality improvement with pre/post implementation | Y | N | Y | Y | Y | N | Y | N | Y | Low |
| A Continued Professional Development Nursing Partnership in a Remote Bolivian Hospital | 2021 | Bolivia | Pre/post quasi experimental | Y | N | Y | N/A | N | Y | Y | Y | Y | Low |
| Effective triage in the Pacific region: The development and implementation of the Solomon Islands Triage Scale. | 2019 | Solomon Islands | Action research methodology; plan, act, observe, reflect cycles | Y | N | Y | N/A | Y | Y | Y | Y | Y | Low |
| The Impact of the Clinical Audit Cycle on the | 2024 | Iran | Pre/post quasi experimental | Y | N | Y | N/A | Y | Y | Y | N | Y | Low |
